# Unveiling the Multilocus Sequence Typing (MLST) Schemes and Core Genome Phylogenies for Genotyping *Chlamydia trachomatis*

**DOI:** 10.3389/fmicb.2018.01854

**Published:** 2018-08-22

**Authors:** Luz H. Patiño, Milena Camargo, Marina Muñoz, Dora I. Ríos-Chaparro, Manuel A. Patarroyo, Juan D. Ramírez

**Affiliations:** ^1^Grupo de Investigaciones Microbiológicas-UR (GIMUR), Programa de Biología, Facultad de Ciencias Naturales y Matemáticas, Universidad del Rosario, Bogotá, Colombia; ^2^Ph.D. Programme in Biomedical and Biological Sciences, Universidad del Rosario, Bogotá, Colombia; ^3^Molecular Biology and Immunology Department, Fundación Instituto de Inmunología de Colombia (FIDIC), Bogotá, Colombia; ^4^School of Medicine and Health Sciences, Universidad del Rosario, Bogotá, Colombia

**Keywords:** MLST, MLST-genotyping, sequence type (ST), schemes, *chlamydia*

## Abstract

Multilocus sequence typing (MLST) has become a useful tool for studying the genetic diversity of important public health pathogens, such as *Chlamydia trachomatis* (*Ct*). Four MLST schemes have been proposed for *Ct* (data available from Chlamydiales MLST databases). However, the lack of a sole standardized scheme represents the greatest limitation regarding typing this species. This study was thus aimed at evaluating the usefulness of the four MLST schemes available for *Ct*, describing each molecular marker's pattern and its contribution toward a description of intra-specific genetic diversity and population structure. The markers for each scheme, showed a variable power of dicrimination, exhibiting in some cases over estimation in the determination of Sequence Types (STs). However, individual analysis of each locus's typing efficiency and discrimination power led to identifying 8 markers as having a suitable pattern for intra-specific typing. analyzing the 8 candidate markers gave a combination of 3 of these loci as an optimal scheme for identifying a large amount of STs, maximizing discrimination power whilst maintaining suitable typing efficiency. One scheme was compared against core genome phylogenies, finding a higher typing resolution through the last approach. These results confirm once again that although complete genome data, in particular from core genome MLST (cgMLST) allow a high resolution clustering for *Ct* isolates. There are combinations of molecular markers that could generate equivalent results, with the advantage of representing an easy implementation strategy and lower costs leading to contribute to the monitoring and molecular epidemiology of *Ct*.

## Introduction

*Chlamydia trachomatis* (*Ct*) has been the species having the greatest clinical and epidemiological importance; it infects the human genital-urinary tract, being the most common bacterial sexually-transmitted infection (STI) worldwide (WHO, 2011; O'connell and Ferone, 2016). Alternatively, *Ct* can infect the ocular mucosa, being responsible for the development of trachoma, the main cause of infectious blindness around the world (Dean et al., 2013; Shao et al., 2013; Anaene et al., 2016; Lallemand et al., 2016). In 2012, the WHO reported around 131 million new *Chlamydia* infections worldwide, the 60 percent of the cases were presented in developed countries, however most of them go unnoticed without diagnosis or adequate treatment (Newman et al., 2015; WHO, 2016). Until now *Ct* constitutes the species with most interest due to the impact on human health (mainly on sexual and reproductive health) (Bom et al., 2011; Christerson and Herrmann, 2012).

*Ct* has a single circular chromosome having more than 1 million base pairs (bp) as well as a 7.5 kb highly conserved plasmid having multiple copies within a cell (Tam et al., 1992; Seth-Smith et al., 2013; Nunes and Gomes, 2014; de Vries et al., 2015; Pawlikowska-Warych et al., 2015; Anaene et al., 2016; Jelocnik et al., 2016). This species is characterized by conserved genomes and by the low level of genetic diversity among variants (< 2% of the genome). However, it presents some regions with high events of recombination and nucleotide diversity (Joseph and Read, 2012). Molecular differences between strains have been seen to be associated with its tropism and geographical distribution; genetically different strains have been identified as infecting various populations [men having sex with men (MSM), heterosexuals and bisexuals] (Gravningen et al., 2012). Such characteristics have led to the use of different typing techniques enabling a strains' tissue tropism to be determined, identifying and differentiating new or persistent infections, understanding transmission dynamics, and monitoring how specific clones evolve (Rawre et al., 2017).

Serotyping has traditionally been used for typing *Ct*; it uses specific antibodies directed against the outer membrane protein (MOMP). However, this technique is considered laborious, takes too long and has low sensitivity (Nunes and Gomes, 2014). Some molecular techniques used for typing *Ct* have been restriction fragment length polymorphism (RFLP), DNA hybridisation-based techniques, polymerase chain reaction (PCR) and DNA microarrays based on analysis of the *ompA* gene (encoding MOMP) (Stothard, 2001; Quint et al., 2007; Pannekoek et al., 2008; Pedersen et al., 2009; Ruettger et al., 2011; Xia and Xiong, 2014; Gallo Vaulet et al., 2016). These have led to 19 variants being identified (Pannekoek et al., 2008), grouped into 3 clusters; one includes variants L1-L3 and L2a, associated with Lymphogranuloma venereum (LGV), another covers variants A, B, Ba, and C, associated with trachoma and another covers variants D-K, Da, Ga, Ia, and Ja, associated with genital-urinary infections (Pedersen et al., 2009; Herrmann et al., 2015; Sherchand et al., 2016; Petrovay et al., 2017).

The low discrimination power of some of the techniques mentioned above and their multiple disadvantages have led these techniques to be replaced by other typing methods especially those based on sequencing, which are much more specific and enable *Ct* intra- specific typing (de Vries et al., 2015). Among these techniques emerges the Multilocus Sequence Typing (MLST) (Klint et al., 2007; Pedersen et al., 2008; Bom et al., 2011; Xia and Xiong, 2014; de Vries et al., 2015) that has provided a portable, reproducible and scalable typing system and is performed easily by different laboratories (Urwin and Maiden, 2003). Additionally, recent studies using whole-genome sequencing (WGS), have allowed expanding the knowledge about the epidemiology, evolutionary history and diversity of members of *Ct* based on recent approaches defined as core genome MLST (cgMLST) (Harris et al., 2012; Rawre et al., 2017). Despite the WGS (Whole genome MLST and cgMLST) has demonstrated to be a tool with a high discriminatory power. This technique presents some disadvantages due to its higher costs and requirement of big computational capacity (Tsang et al., 2017; Versteeg et al., 2018).

Several MLST schemes have been described to date for genotyping *Ct* and have been designed with different purposes (Supplementary Table S1); one has been designed to analyse evolutionary changes over time and its usefulness for comparison of strains from different species (Pannekoek et al., 2008) and others for describing *Ct* intra-taxa variability (Dean et al., 2009), one such based on seven housekeeping genes (*C. trachomatis* MLST scheme) and another on five highly variable regions (*C. trachomatis -* Uppsala MLST scheme) (Grieshaber et al., 2006; Klint et al., 2007) has been designed to discriminate only *C. trachomatis* strains for epidemiological purposes. Finally, there is also the plasmid loci MLST scheme (https://pubmlst.org/chlamydiales/), which makes use of regions in the 8 putative open reading frames encoded by a 7.5 Kbp plasmid in most *Ct* isolates (Rockey, 2011), however there is not enough information associated with its use.

In spite of MLST schemes' many advantages and clinical applications, there is currently no single standardized scheme for typing *Ct*. This study was thus aimed at analyzing all the MLST schemes available for *Ct* (Chlamydiales, *C. trachomatis, C. trachomatis—*Uppsala and plasmid loci) to determine the schemes' robustness (resulting from combining multiple loci), as well as the molecular markers independently, and evaluate their usefulness for describing intra-specific genetic variability. It was also aimed at evaluating how such information can describe *Ct* genetic population structure, representing an indicator of transmission dynamics and signals leading to the variability of this group of organisms. We finally compared the results with phylogenies retrieved from cgMLST (Tsang et al., 2017). The study sought to identify the best combination of molecular markers enabling *Ct* isolate typing, maintaining suitable intra-species discrimination power using a core genome MLST as reference. Finally, it is important to mention that although the data obtained in each scheme come from different studies and were performed with different purposes, they represent to date the dataset currently available worldwide for *Ct*.

## Materials and methods

### Data retrieval

All data were obtained from public databases for molecular typing and microbial genome diversity (https://pubmlst.org/) (Jolley and Maiden, 2010); such databases (curated and public access) included the Chlamydiales MLST website where descriptive data set was accessed (related to geographical origin, characteristics regarding source and traditional classification, etc.), isolates reported to date (https://pubmlst.org/bigsdb?db=pubmlst_chlamydiales_isolates) as well as downloading sequences covering all known diversity for the Chlamydiales species and *Ct* variants via the locus/sequence definitions database (https://pubmlst.org/bigsdb?db=pubmlst_chlamydiales_seqdef).

The date of the last update of the database at the moment of conducting the analyzes was 03-15-2017.

**Figure 1:**
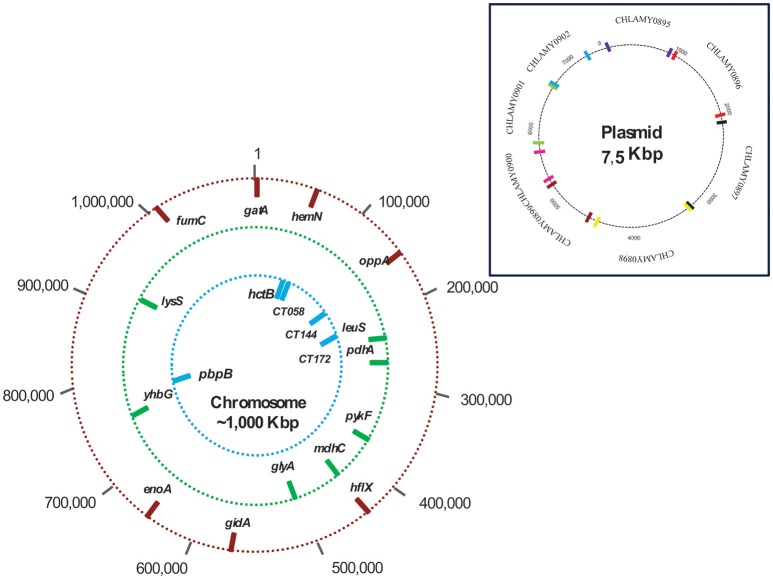
Chromosome and plasmid mapping of studied loci. The outer circle (red bars) shows the location of the 7 markers used in the scheme A (Chlamydiales-*Ct*); the middle circle (green bars) shows the location of the 7 markers in scheme B (*C. trachomatis*); and the inner circle (blue bars) shows the location of the 5 markers in scheme C (*C. trachomatis* Uppsala). Scheme D molecular markers are plasmid loci.

Four MLST schemes were found in the Chlamydiales MLST database, the first includes the genes *gatA, oppA, hflX, gidA, enoA, hemN*, and *fumC* (Pannekoek et al., 2008). This scheme is the most used for isolates of the order Chlamydiales, because it allows discrimination at the species level. However, when considering the objective of the present work, exclusively the sequences of *Ct* were selected and used for the analysis conducted for the group that is referred to as Scheme A. The three remaining schemes focus exclusively on the typing of *Ct* isolates: Scheme B *C. trachomatis* MLST scheme (*glyA, mdhC, pdhA, yhbG, pykF, lysS*, and *leuS*) (Dean et al., 2009), Scheme C *C. trachomatis -* Uppsala MLST scheme (*CT058, CT144, CT172, hctB, and pbpB*) (Klint et al., 2007) and Scheme D the plasmid loci MLST scheme (CHLAM0895, CHLAM0896, CHLAM0897, CHLAM0898, CHLAM0899, CHLAM0900, CHLAM0901, and CHLAM0902). The seven genes used in Schemes A,B were housekeeping genes, whilst Scheme C's five genes were considered hypervariable. The four MLST schemes made use of 27 molecular markers constituting the dataset for subsequent analysis. Figure 1 and Supplementary Table S1 give information regarding the genes included in the MLST schemes.

### Descriptive analysis

Chlamydial species' descriptive analyses were conducted from the breakdown section's exportable dataset (https://pubmlst.org/bigsdb?db=pubmlst_chlamydiales_isolates&page=job&id=BIGSdb_057458_1492173438_56586). This dataset gave information/variables regarding the hosts from which each isolate was obtained concerning age, country, region, sample source, gender, host and *Ct* variant isolated. Variables were treated as categorical and described in terms of frequency and percentage; 95% confidence intervals were used when events of interest were calculated (bootstrap). Chi^2^ or Fischer's exact tests were used for estimating differences regarding distribution, depending on the nature of the data. STATA12® software was used for all statistical analysis (0.05 significance for all hypothesis testing).

### Molecular markers characteristics

The allele sequences reported for each molecular marker were downloaded in FASTA format via the option, ‘Download allele sequences' (https://pubmlst.org/bigsdb?db=pubmlst_chlamydiales_seqdef&page=downloadAlleles&tree=1), providing alignments for the different schemes available. The sequences for all allele profiles reported for each MLST scheme were downloaded via the option, “Export allele sequences in XMFA/concatenated FASTA formats—Chlamydiales locus/sequence definitions” (https://pubmlst.org/bigsdb?page=plugin&name=SequenceExport&db=pubmlst_chlamydiales_seqdef), selecting all loci included in the MLST scheme. The ClustalW algorithm was used for initially comparing all sequences via multiple alignment (Thompson et al., 2002) to identify the percentage of identity regarding alleles' total length.

Each marker's nucleotide composition was then analyzed for identifying the amount of variable sites between the reported alleles and compared with those sites considered as informative according with parsimony principle (positions containing at least two types of nucleotides having a minimum frequency of two). This rate, named here “informative indices” was calculated to provide comparable data regarding the molecular markers.

### Genetic diversity indices

The sequences were aligned using MUSCLE (alleles reported for each marker and total STs concatenated sequences), later were evaluated to identify insertion and deletion (indels) events, which might have altered the length of the sequences to be analyzed. Once the indels were identified, those were edited to eliminate the gaps. DnaSP software (v5) was then used for analyzing verified alignments' genetic diversity: total amount of mutations (Eta), amount of haplotypes (h), haplotype diversity (Hd), defined as the probability that two randomly chosen haplotypes would be different, nucleotide diversity (π), representing the average number of nucleotide differences per site between two randomly chosen DNA sequences, Theta (per site) from Eta, Theta (per site) from S (ThetaW), where Eta (h) represented the total amount of mutations and S was the amount of segregating (polymorphic) sites and average number of nucleotide differences (k). Some calculated indices [Haplotype diversity and Theta (per site) from S (ThetaW)] are reported with their respective 95% confidence intervals.

### Phylogenetic analyses

Phylogenetic reconstructions were made from the alignments regarding the total length of molecular markers included in each MLST scheme (for each gene and concatenated sequence for each scheme). The jModelTest v.2.1.10 (Lemey, 2009; Darriba et al., 2012) was applied on all the alignments, considering the Akaike Information Criterion “AIC” (Alfaro and Huelsenbeck, 2006), in order to identify the best substitution model for phylogenetic reconstructions. Considering that Tamura-nei (TrN) (Tamura and Nei, 1993) was the model that consistently presented low AIC results, this was applied for all subsequent analyses.

Bootstrap method (BT; with 1,000 replicates) was used for evaluating the nodes' robustness, considered a well-known statistical tool for approximates of the variance of the data under the real model of sequence evolution (Wróbel, 2008). Each phylogenetic reconstruction was analyzed for identifying the number of clusters produced, defined as nodes having ≥80.0% BT values. A second screening was necessary for molecular markers where no clusters exceeding this cut-off point were identified, reducing ≥60.0% bootstrap cut-off values. BT replicates were increased to 10,000 when ≤1 cluster was identified, during the preliminary phylogenetic analysis (i.e., Scheme B). It has classically been reported that more than 1,000 replicates are needed to attain ±1% accuracy for bootstrap proportions of 95% or higher (Hedges, 1992). Increasing the number of BT replicates would produce greater resolution power, contributing in the evaluation of statistical significance of the relative validity of phylogenetic reconstructions (Müller, 2005; Deng et al., 2013). Homologous genes in closely related species were identified for each marker as outgroup for the phylogenetic reconstructions. Homologous genes in *Parachlamydiaceae acanthamoebae* were used in Schemes A,B and homologous regions in *C. muridarum* were included in Schemes C,D. FastTree version 2.1.9 Double precision (Price et al., 2010), was used to conduct phylogenetic trees based on molecular markers.

ST classification for each marker/scheme was graphically represented via allele plot, assigning a color to each well-supported cluster in each phylogenetic reconstruction. It was then determined to which each ST belonged (assigning a corresponding color). The number of colors in allele plots thus represented the amount of clusters discriminated by each molecular marker as reported elsewhere (Muñoz et al., 2017). Molecular Evolutionary Genetics Analysis software (MEGA7 version 7) was used for all alignments and phylogenetic reconstructions (Kumar et al., 2016).

### Multilocus sequence analysis (MLSA)

The allele profiles for the STs reported for each MLST scheme were analyzed to identify related ST groups (clone complexes—CC) and make evolutionary inferences by identifying founder genotypes (ST) for each CC identified via each MLST scheme; eBURSTv3 software was used for such analysis (Feil et al., 2004). Parallel to this, phylogenetic networks were developed using the Neighbor-net method available in the SplitsTree4 package (version 4.14-4) for identifying rearrangements to which the molecular markers included in each MLST scheme (loss and duplication events, hybridisation, horizontal gene transfer or recombination) could undergo (Huson and Bryant, 2006).

### Comparison of MLST schemes with whole genome sequencing data

An additional analysis was conducted using WGS data, considering it as the most robust source of data to evaluate the clustering of isolates and to plausibly depict the best-fit *Ct* typing Scheme. A set of public available genomes was downloaded and then used to compare the clustering obtained by the multiple MLST schemes against wgs typing. The data set was obtained from the following databases: PATRIC 3.5.11 (https://www.patricbrc.org/view/GenomeList/?and(keyword(chlamydia),keyword(trachomatis))#view_tab=genomes), NCBI Sequence Read Archive—SRA (https://trace.ncbi.nlm.nih.gov/Traces/sra/sra.cgi?view=announcement), European Nucleotide Archive—ENA (https://www.ebi.ac.uk/ena/data/search?query=chlamydia+trachomatis), National Center for Biotechnology Information (NCBI) Reference Sequence (RefSeq) database (http://www.ncbi.nlm.nih.gov/refseq/) and Wellcome Sanger Institute (http://www.sanger.ac.uk/resources/downloads/bacteria/chlamydia-trachomatis.html). All databases consulted are curated and freely accessible.

“*Chlamydia trachomatis*” was used as a search term in the different databases. For each match found, the multi-file assembly was downloaded. For the genomes found in more than one database, only one report was considered. Once the complete genomes were obtained, the quality control of the raw data was carried out using the GenomeQC Filter (v1-5.pl), which considers the following parameters: (i) a maximum number of 400 contigs allowed, (ii) a maximum genome size of 8 Mb, and (iii) a similarity of at least 95% between 16S ribosomal RNA (16SrRNA) sequences. The genomes with poor quality were excluded. In parallel, the extracted 16S rRNA sequence was used, both for the verification of taxonomic allocation using the SINA Alignment Service tool, available in SILVA rRNA gene database (Quast et al., 2013), SILVA database, and for the generation of a phylogenetic reconstruction based on 16SrRNA, in order to verify the clustering within the same species.

The genomes that passed the quality tests were used to predict the ST considering the Chlamydiales scheme, using the mlst-2.10 package (Seemann, 2018). This tool was used to predict the allelic profiles of the set of genomes evaluated, both by Scheme A, which is predetermined within the databases included in the mlst-2.10 package, and by Schemes B,C, which were added to the databases, using the information available in Chlamydiales MLST database. The concatenated sequence of the determined ST was used to construct a multiple alignment and to carry out a phylogenetic reconstruction.

In parallel, the set of selected genomes was annotated using Prokka version 1.13 (Seemann, 2014), as a preliminary step for determining the pangenome of the analyzed data set using Roary (by means of a blastp percentage identity of 95% and a core definition of 99%) (Page et al., 2015). A phylogenetic tree based on the core genome of the analyzed data set was inferred, which was considered as a '**reference**' of the clustering ('core genes' are shared by more than 95% of the data included in the analysis and represent the most robust data set for the generation of high resolution phylogenies) (Sentausa and Fournier, 2013; Wang et al., 2015). Additionally, the multi FASTA alignment file of core genome was used to identify the Single Nucleotide Polymorphisms, using the SNP-sites program (Page et al., 2016). Phylogenetic reconstructions from core genome SNPs were conducted to compare the clustering of the schemes against the markers herein evaluated. For phylogenetic reconstructions based on core genome and core genome SNPs, the alignments were analyzed using the Randomized Axelerated Maximum Likelihood (RAxML v.8) method. The clustering of the set of isolates was then evaluated through the comparison of the obtained phylogenetic reconstructions.

### Marker usefulness for intra- specific typing

MLSTest software was used for calculating the number of alleles and polymorphisms, typing efficiency (TE) and discriminatory power (DP), using Simpson's index (and 95% IC) (Tomasini et al., 2013). Each marker's alignment was used as data source, including alleles for all STs reported here. MEGA7 software (Nei and Gojobori's method) was used for calculating the ratio of non-synonymous (dN) to synonymous (dS) substitutions per nucleotide site (dN/dS) for inferring the type of selection to which each molecular marker was exposed. TE and DP were described in terms of means and standard deviations (SD) for the Schemes. Markers having high TE and DP (within the 75 percentile) were then analyzed regarding scheme optimisation for identifying the optimum number of loci required. Such analysis involved the sequences for 179 isolates constituting the only group having information for the 3 MLST schemes (Schemes A–C), considering that Scheme D is used *in silico*, was excluded from this analysis.

## Results

### Descriptive analysis

Initial analysis of the Chlamydiales MLST database revealed information available for 4,024 isolates, including those having a typing result by any of the 4 MLST schemes evaluated here. The aforementioned isolates had been recorded from 1957 to 2017 and had been reported in the database up to 2017-03-15 (last update taken for data analysis). Of the total data reported, 3,691 correpond to *Ct*, this data set was used for describing distribution profiles for *Ct* (for clusters, variants or STs), according to age, gender and/or sample source.

Geographical distribution pattern of *Ct* was analyzed regarding a set of 3,133 data set isolates; 78.1% (*n* = 2,448) of the isolates were reported in Europe (Austria, Denmark, France, Germany, Norway, Poland, Portugal, Spain, Sweden, Switzerland, the Netherlands and the United Kingdom), 8.8% (*n* = 277) from South America (Argentina, Chile, Ecuador and Suriname), 5.4% (*n* = 168) from Africa (South Africa, Tanzania, the Gambia and Tunisia), 4.1% (*n* = 129) from Asia (China, Nepal, Russia, Saudi Arabia and Taiwan), 3.4% (*n* = 105) from North America (Canada and the USA), and 0.2% (*n* = 6) from Oceania (Australia). The highest percentage of isolates was reported from the Netherlands (57.2%; *n* = 1,793), followed by Sweden (11%; *n* = 342), and Norway (7.9%; *n* = 249). The remaining countries had < 5% isolates (Supplementary Figure S1). As most available data concerned *Ct*, some characteristics of interest for this species were described, i.e., gender, sample source, variant, worldwide distribution, sampling site of the clusters analyzed and age (Supplementary Table S2 and Supplementary Figure S2).

### Describing the MLST schemes

The Chlamydiales MLST Databases contained 16,019 sequences at cut-off date. This included those used for classifying each MLST scheme and led to identifying 75 STs for Scheme A, 44 STs for Scheme B, 520 STs for Scheme C, and 47 STs for Scheme D. Regarding the amount of STs described for each MLST scheme for each *Ct* variant, there were more Cluster 2 (associated with genitourinary infections) variants in all schemes, contrasting with that observed for variants related to LGV (including the least amount of STs) (Supplementary Table S3 and Supplementary Figure S3). Supplementary Figure S3 describes the amount and frequency of each ST per variant, discriminating classification by each MLST scheme.

Initial descriptive analysis of the sequences reported for each molecular marker led to determining that the number of alleles reported ranged from 7 (for *glyA, pdhA*, and *pykF* genes used for Scheme B) to 92 (*hctB* used for Scheme C). Sequence identity analysis of alleles reported for each molecular marker showed that Scheme A shows identity percentages between 39.2 and 99.1% (being *hemN* the one that showed less percentage of identity and *gatA* the one with the highest one). Greater than 95% identity was observed for all genes in Scheme B whilst markers in Scheme C were highly heterogeneous, ranging from 14.7% (marker CT172) to 88.6% (CT086). Identity values ranging from 83.6% (marker CHLAM0895) to 98.3% (CHLAM0897) were found for Scheme D. Sequence identity percentages can be consulted in Supplementary Figure S4-A.

Considering that not all variable sites were informative (according to parsimony principle), the rate between the number of variable sites vs. informative sites was calculated, with the aim of generating a comparative data (named “informative index” here) between molecular markers. The results were inversely proportional to sequence identity patterns (genes having the highest identity percentages displayed the lowest informative indexes). The most interesting informative index pattern was for Scheme D, in which markers CHLAM0895 and CHLAM0898 had the maximum result (1.0000), indicating that all variable sites could be considered as informative, contrary to what happened with marker CHLAM0900, that showed a null result (0.000), provided none of the variable sites were informative. Supplementary Figure S4-B describes the number of variable sites compared to informative ones for all molecular markers.

### Analyzing genetic diversity

Supplementary Table S4 reports the intra-species genetic diversity indices calculated for each MLST scheme (molecular markers and concatenated sequences). Figure 3 gives a graphical representation of nucleotide diversity compared to haplotype diversity indices for each set of data. Nucleotide diversity values were < 0.2222 for all schemes concerning independent analysis for each marker and concatenated sequence. However, when comparing MLST schemes, the results showed that the highest nucleotide diversity indices were present in Scheme C, marker CT172 (0.18962) and concatenated sequences (0.22224). The rest of the markers showed nucleotide diversity <0.0568.

The greatest nucleotide diversity for Scheme B was 0.03129 (*hemN*) and 0.00950 (CHLAM0899) for Scheme D. Regarding haplotype diversity, it was found that Schemes A,B,D had values close to 1.000, differently to Schemes A,C where heterogeneous patterns were observed, being lower for Scheme A [0.328 (*hemN*) to 0.771 (*gidA*)] compared to Scheme C [0.801 (CT172)]. Supplementary Table S4 gives all genetic diversity indices calculated for each MLST scheme.

### Analyzing clonal complexes (CC)

The concatenated sequences for each MLST scheme evaluated were used for depicting CC clustering patterns via the eBURST algorithm. The results showed that the STs identified via Scheme A led to 3 CC and 6 singletons being identified; CC1 and CC2 had most STs (36 and 26 STs, respectively), their founder STs (ST13 and ST4, respectively) being associated with urogenital infections. Founder CC3 (including 7 ST) was associated with LGV (ST44).

eBURST analysis of Scheme B, 3 CC and 11 singletons were identified; CC1 had most STs (19 STs), founder ST 19 being associated with urogenital infections, followed by CC2 and CC3 having the same amount of STs (7) and whose founder ST 11 has been associated with urogenital lesions and 34 with trachoma. Regarding Scheme C was grouped into 15 CC and 55 singletons, having two CC mainly consisting of 241 and 167 STs, respectively. Founder ST for these majority groups were 56 and 106, both being associated with urogenital infections. When evaluating Scheme D, 6 CC and 12 singletons were identified, CC1 having most STs (12 STs) which were associated with urogenital lesions. Figure 4 describes most of CC organization for each MLST scheme. Supplementary Table S5 gives complete eBURST analysis results for each MLST scheme.

### Clustering each MLST scheme/molecular marker

Phylogenetic reconstructions of the sequences for all STs reported for each MLST scheme were made for each molecular marker and concatenated sequence; they were then used as the basis for determining their discrimination power (represented in allele plot, Figure 5). Regarding the discriminatory power, the results showed that Scheme A identified 2–4 well-supported clusters, and the marker *hemN* providing most clusters.

Analyzing Scheme B highlighted all markers' (1–4 cluster) low discrimination power, the *mdhC* gene being the marker having the greatest discrimination power (the only one having 4 clusters). These results led to identifying these markers' low polymorphism. It was found that Scheme C had high discrimination power; 20 well-supported clusters and 3 outliers were found in phylogenetic reconstruction based on concatenated sequences. This pattern was confirmed by grouping by markers such as CT144 (12 clusters) and pbpB (7 clusters). Interestingly, it was found that marker CT172 only produced 1 cluster which included all the STs.

Analyzing Scheme D led to determining the high discrimination power of this scheme's loci (3-8 clusters), loci CHLAM0895, CHLAM0898, and CHLAM0899 being the markers having the greatest discrimination power.

The allele plot patterns showed less clusters (less diversity of colors). These were observed for schemes directed toward *Ct* typing, especially Scheme B, where a single cluster (in green) predominated, followed by Scheme A, where only some STs belonged to a second cluster with relative frequency (in red). In contrast, the Scheme D was the only MLST scheme having a pattern displaying many clusters (represented by the greatest diversity of colors) (Figure 5).

SplitsTree software was used for constructing phylogenetic networks to verify molecular rearrangements regarding the molecular markers used in each scheme (Neighbor-net algorithm). Concatenated sequence analysis for each scheme revealed reticulation events, mainly for Scheme C. In spite of preliminary indications of recombination identified for Scheme D, no reticulation events were found in the allele plot for the phylogenetic network. Finally, no marked reticulation events were observed for Schemes A,B (Supplementary Figure S5).

### WGS phylogenetic reconstructions' comparison

In total, 243 complete genomes were found in the different databases consulted. The quality control analyses led to the exclusion of 83 genomes, because 13 of them showed a contig count above the established limit (between 419 and 15,664 contigs) and in the remaining 70 genomes, the 16SrRNA sequence was not identified. The additional step for verifying the taxonomic allocation of the set of genomes using the SINA Alignment Service tool, available in the SILVA rRNA gene database (Quast et al., 2013), showed that one of them corresponded to *Mycoplasma* (BioSample Accession: SAMEA1398231). This finding was confirmed in the phylogenetic reconstruction based on 16SrRNA that was carried out in parallel, where the sequence of this genome (813.61), was the only one that clustered outside the *Ct* cluster (Supplementary Figure S6).

Finally, a set of 158 *Ct* genomes were subjected to ST identification, using the three MLST schemes targeting chromosomal genes. We identified 19 STs using Scheme A, 15 STs by Scheme B and 26 STs by Scheme C. Interestingly, allelic profiles and alleles that had not been reported in Chlamydiales MLST database were found within the data set, corresponding to 20 genomes for the case of Scheme A, 68 for Scheme B and 72 for Scheme C. The concatenated sequences for the seven house keeping genes of the Chlamydiales scheme were extracted and used to conduct multiple alignments and the subsequent phylogenetic reconstruction. The results showed reduced clustering discrimination by A and B schemes without evidence of clustering according to tropism (Figures 6A,B). In the case of Scheme C (Figure 6C), although clearer clustering profiles were identified, the topology of the tree did not allow a clear clustering by topology.

Considering that the core genome could be more informative in the evolutionary context. The annotated genomes (using prokka) were used to depict the *Ct* pangenome. A total of 3,177 genes defined the pangenome of the analyzed data set, of which 794 corresponded to the core genome. These genomes were used to perform the subsequent phylogenetic reconstructions (core genome phylogeny and core genome SNP phylogeny). When analyzing the data obtained in the core genome phylogeny, we observed the emergence of four clusters, whose sequences were mostly grouped according to their tropism (mainly those associated with ocular infections) (Figure 6D). This behavior was the same when performing the SNP core genome phylogeny (Figure 6E).

### Typing efficiency and discrimination power for 27 loci

Regarding typing efficiency (TE) (Table 1), the best results were obtained for Scheme B (1.0386 average) whose genes had >0.889 TE, except for *yhbG* (0.381). Analysis of Scheme A revealed increased TE for most genes (reaching an average of 0.7928); reduced TE was observed regarding *gidA* and *hemN*, the latter having the lowest value amongst the 27 markers evaluated here (0.026). The genes included in Scheme D had 0.7344 average TE; the gene having the greatest TE amongst the 27 molecular markers evaluated here was included in this scheme: *CHLAM0895* (1.500). Average ET for Scheme C was 0.3432, CT058 being the marker giving the best result (0.750).

**Table 1:** Calculating the typing efficiency and discriminatory power of the markers in the schemes analyzed.

**MLST scheme**	**Molecular marker**	**Typing efficiency**	**Mean per scheme [SD]**	**Discriminatory power [95% CI]**	**Mean per scheme [SD]**	**dN/dS**
Scheme A	*gatA*	1.250	0.7928 [0.5305]	0.668 [0.586–0.751]	0.5841 [0.1741]	0.0094
	*oppA*	1.111		0.624 [0.516–0.732]		0.0191
	*hflX*	1.250		0.708 [0.634–0.782]		0.0184
	*gidA*	0.134		0.771 [0.717–0.826]		0.0200
	*enoA*	1.143		0.639 [0.56–0.718]		0.0184
	*hemN*	0.026		0.328 [0.191–0.464]		0.5880
	*fumC*	0.636		0.351 [0.211–0.492]		0.0172
	Combination of loci	0.148		1 [1–1]		
Scheme B	*glyA*	1.400	1.0386 [0.3401]	0.538 [0.372–0.705]	0.5534 [0.1918]	0.0096
	*mdhC*	1.333		0.289 [0.108–0.469]		0.0058
	*pdhA*	1.167		0.331 [0.139–0.523]		0.0109
	*yhbG*	0.381		0.526 [0.342–0.711]		0.0417
	*pykF*	1.000		0.739 [0.647–0.831]		0.0133
	*lysS*	0.889		0.668 [0.527–0.809]		0.0156
	*leuS*	1.100		0.783 [0.671–0.895]		0.0193
	Combination of loci	0.852		1 [1–1]		
Scheme C	CT058	0.75	0.3432 [0.2579]	0.875 [0.86–0.89]	0.8774 [0.0404]	0.0904
	CT144	0.277		0.829 [0.811–0.848]		0.2288
	CT172	0.092		0.848 [0.824–0.873]		0.6207
	*hctB*	0.178		0.925 [0.909–0.941]		0.0785
	*pbpB*	0.419		0.91 [0.903–0.918]		0.1429
	Combination of loci	0.183		1 [1–1]		
Scheme D	*CHLAM0895*	0.565	0.7344 [0.3559]	0.795 [0.708–0.881]	0.7759 [0.0252]	0.0251
	*CHLAM0896*	0.611		0.776 [0.675–0.878]		0.0181
	*CHLAM0897*	0.667		0.87 [0.826–0.913]		0.0133
	*CHLAM0898*	0.333		0.887 [0.841–0.933]		0.0201
	*CHLAM0899*	0.522		0.865 [0.819–0.91]		0.0289
	*CHLAM0900*	1.500		0.303 [0.131–0.476]		0.0075
	*CHLAM0901*	0.867		0.825 [0.759–0.891]		0.0189
	*CHLAM0902*	0.81		0.886 [0.837–0.935]		0.0282
	Combination of loci	0.586		1 [1–1]		

Evaluating discriminatory power (DP) (Table 1) revealed that Scheme C gave the highest results (0.8774 average) and included the genes having the highest result for all markers *hctb*: 0.925 and *pbpB*: 0.91. The Scheme D (0.7759 average) was the other scheme having high average DP; the markers giving the best results for these schemes were *CHLAM0898* (0.887) and *hctB* (0.925). Schemes A (0.5841) and B (0.5534) had the lowest DP. The marker having the lowest DP amongst the 27 genes was *mdhC* (0.289) from Scheme B.

Comparing TE and DP results for all markers revealed that only 8 markers had both results within the 75th percentile (Table 2). These markers' pattern was analyzed regarding the set of 179 isolates for which information was available for all MLST schemes. It was found that TE ranged from 0.883 (*leuS*) to 0.286 (*CHLAM0895*) and DP from 0.808 (*CT058*) to 0.603 (*leuS*).

**Table 2:** Typing efficiency and discriminatory power of the markers herein selected.

**MLST scheme**	**Molecular marker**	**Typing efficiency**	**Discriminatory power (95% CI)**
Scheme A	*gidA*	1.167	0.718 (0.684–0.751)
Scheme B	*leuS*	1	0.384 (0.299–0.468)
Scheme C	*LysS*	1	0.312 (0.227–0.398)
	*CT058*	0.517	0.823 (0.789–0.857)
	*CT172*	0.056	0.879 (0.849–0.908)
	*hctB*	0.052	0.843 (0.803–0.883)
	*pbpB*	0.194	0.874 (0.857–0.891)
Combination of loci		0.139	0.976 (0.969–0.984)

After performing, a comparison with a “true scheme” as cgMLST and observing in general similar results with the compared schemes (MLST schemes herein evaluated). We decided to observe the plausibility of optimizing an adequate MLST scheme with the available genes. Scheme optimisation revealed that the optimum number of loci required for identifying the largest amount of STs was 5; combinations of genes led to 69 STs being identified in this set of isolates. Even though 6 or more genes were included, a maximum of 72 STs were identified (Supplementary Table S6); *CHLAM902* gave the best TE and DP results for any of the last three markers (Table 2), meaning that this combination can be proposed as the optimum combination of markers for classifying this dataset. The harmonized proposal arises from analyses carried out here and is shown in Figure 7.

The concatenated sequences for all STs reported in each MLST scheme were used for the extraction of both SNPs and Pi sites, which were then analyzed for the usefulness of the MLST tool by determining the TE and DP (with their corresponding 95% CI), as described previously. These findings were compared with the results obtained from the complete sequences (Table 3), finding that the number of alleles identified for each MLST scheme from SNPs is almost equal to that of the complete sequence. Since, it is precisely these variations the characters informative by this approach, however the number of alleles identified from Pi sites is reduced. For the case of TE and DP, the results are similar, in the case of Schemes B,C. The Pi sites show higher TE with respect to that determined from SNPs. In the case of Schemes A and D, the TE was reduced to almost half in the case of Pi sites, regarding the SNPs.

**Table 3:** Calculation of SNPs and Pi sites according to the scheme evaluated.

	**Scheme A**			**Scheme B**			**Scheme C**			**Scheme D**		
	**Complete sequence**	**SNPs**	**Pi sites**	**Complete sequence**	**SNPs**	**Pi sites**	**Complete sequence**	**SNPs**	**Pi sites**	**Complete sequence**	**SNPs**	**Pi sites**
Number of alleles	75	74	53	44	44	37	520	501	454	47	46	16
Number of polymorphisms	435	223	198	61	61	45	1747	665	520	161	140	93
Typing efficiency	0.172	0.332	0.268	0.721	0.721	0.822	0.298	0.753	0.873	0.292	0.329	0.172
DP (95% confidence interval)	1 (1–1)	1 (0.999–1)	0.986 (0.977–0.995)	1 (1–1)	1 (1–1)	0.99 (0.98–1)	1 (1–1)	1 (1–1)	0.999 (0.999–1)	1 (1–1)	0.999 (0.996–1)	0.913 (0.877–0.949)

## Discussion

Appropriate identification of *Ct* variants enables understanding infection's transmission dynamics and natural history. Different techniques have been developed for such purpose, including MLST, known for its high-resolution power and producing useful data for describing population structure at epidemiological, genetic and/or evolutionary levels (de Vries et al., 2015). There is a secure, open-access database for Chlamydiales (Chlamydiales MLST database https://pubmlst.org/chlamydiales/) which has information regarding the isolates obtained from different parts of the world and also contains sequences from different schemes, including the four MLST schemes (Jolley and Maiden, 2010; Maiden et al., 2013). The different typing schemes evaluated have been developed for different purposes (analysis of evolutionary changes, discrimination between strains and epidemiological analyzes). However, it is necessary to find a limited number of MLST markers that provide the best discriminatory power that can subsequently be employed within a single, effective and efficient scheme that can be used globally.

**Figure 2:**
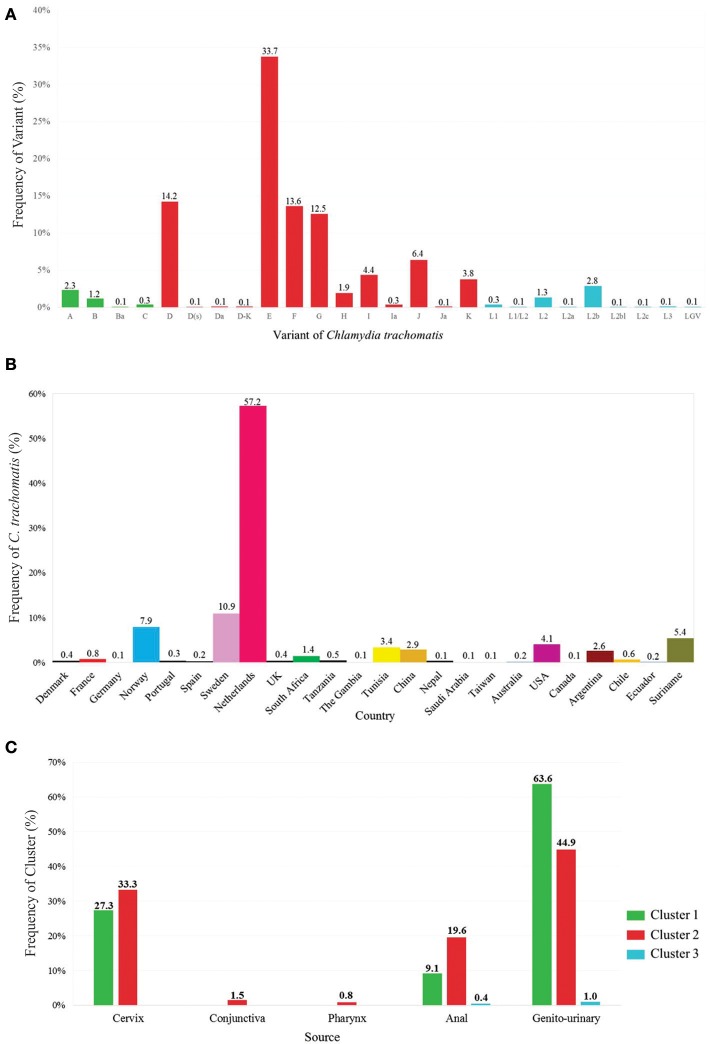
Description of *Ct* variants in isolates reported in Chlamydiales MLST databases. **(A)** *Ct* variant frequency (*n* = 3,242): Green shows cluster 1 variants (associated with ocular infections), red shows cluster 2 variants (associated with urogenital infections) and blue shows cluster 3 variants (associated with *Lymphogranuloma venereum*). **(B)** *Ct* distribution according to country (*n* = 3,242). **(C)** *Ct* cluster distribution according to sample source (*n* = 2,194).

The results obtained in these databases allowed us to conduct a descriptive analysis of the information contained therein and althought the original data is not population based, allowed us to compare them with was currently reported in the literature. One of these analyzes allowed us to determine that variants E, D, F, and G (included in Cluster 2) occurred most frequently regarding the 19 variants currently known for *Ct* (Figures 2A–D); this agreed with that reported previously, where close to 50% of genital tract infections caused by *Ct* were associated with such variants (Nunes et al., 2010; O'connell and Ferone, 2016). On the other hand, it has been observed that *Ct* variants are grouped according to three types of pathology and tissue tropism (ocular, urogenital and LGV). However and interestingly, the results obtained from the database indicated that variants such as those belonging to Cluster 1 (reported as being related with ocular tropism) had been exclusively isolated from genitourinary samples, which can partly explain why *Ct* variants were able to colonize differing ecological niches (Harris et al., 2012) (Ferreira et al., 2014). Finally, we observed that around 95% of the isolates included in the database were from individuals aged 16–29 years-old, thereby agreeing with the information reported by the Centers for Disease Control and Prevention, which has described that most cases associated with *Chlamydia* are presented in adolescents and young adults (Lagkouvardos et al., 2014; O'connell and Ferone, 2016). The currently available epidemiological and clinical data has revealed high *Ct* infection prevalence worldwide; such information has led to the development of screening and molecular typing methods for evaluating the impact of infection by Chlamydiales species and broadening knowledge concerning its genetic and population structure (Gharsallah et al., 2016; Versteeg et al., 2016). Analyzing the four MLST schemes available for *Ct* has led to identifying characteristics related to inter- and intra-taxa discrimination power.

**Figure 3:**
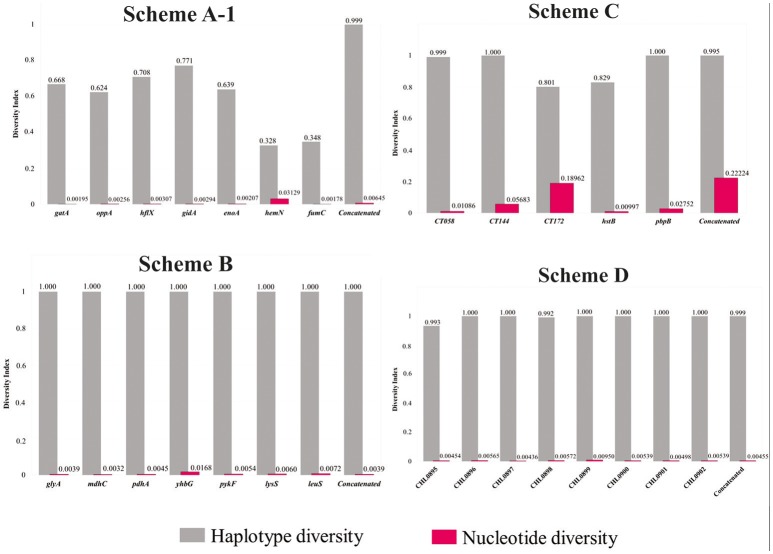
Haplotype diversity (Hd) and nucleotide diversity (pi) indexes for each marker and concatenated sequence for every MLST scheme. DnaSP software was used for calculating the indices.

**Figure 4:**
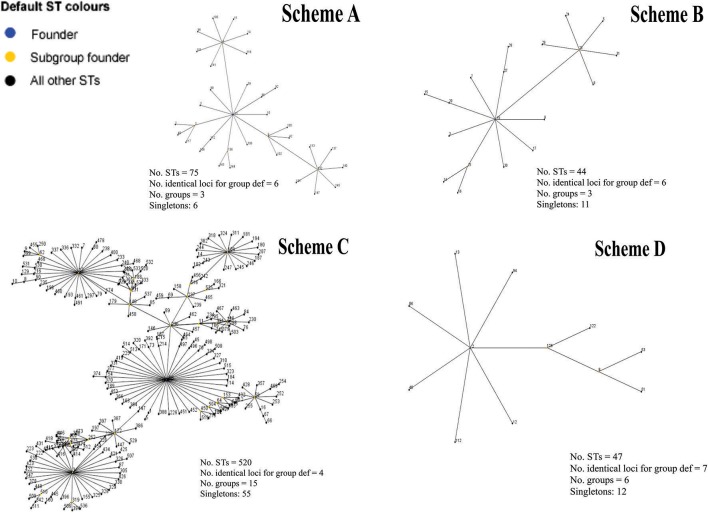
Diagram of the main clonal complexes identified by eBURST analysis. Graphical representation of the clonal complexes, including the greatest amount of STs for each scheme evaluated. Yellow color shows the ST founder and blue color the subgroup founder.

**Figure 5:**
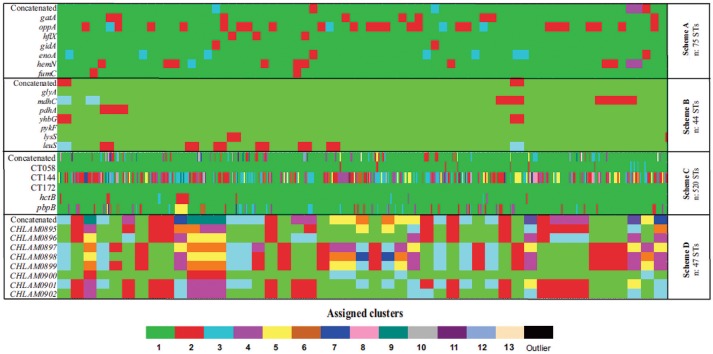
Allele plot. Graphical representation of phylogenetic inferences regarding the markers for the MLST schemes evaluated; each cluster is represented by a color (≥80.0% bootstrap) found per gene (row). The cluster to which each ST (columns) belongs was analyzed. **(A)** Scheme A (Chlamydiales-*Ct*). **(B)** Scheme B (*C. trachomatis*), **(C)** scheme C (*C. trachomatis* Uppsala), **(D)** scheme D (plasmid loci).

Regarding Scheme A (based on housekeeping genes and directed toward inter-species identification), the results showed that all its genes had a low percentage of identity and nucleotide diversity index (Figure 3), this being the only scheme where it was observed that only one marker (*hemN*) had a large number of informative sites (>0.9) (Supplementary Figure S4). Concerning population structure analysis, a short amount of CCs was observed (*n* = 3) (Figure 4), this result was confirmed by the low number of clusters produced in the allele plot (Figure 5) and few reticulation events in phylogenetic networks (Supplementary Figure S5). The results suggested that their use in intra-species discrimination would be debatable.

Regarding intra-species identification, Scheme B (also based on housekeeping genes), it was found that all the genes had a high percentage of identity, reduced number of informative sites (Supplementary Figure S4) and low nucleotide diversity indices, thereby indicating their conserved nature (Figure 3). Concerning population structure, it was found that 3 CCs grouped most STs reported here (*n* = 33/44) (Figure 4), this being confirmed by the uniformity regarding STs discrimination, described by the reduced number of clusters in the allele plot (Figure 5) and the limited amount of reticulation events in the phylogenetic networks (Supplementary Figure S5). These results would thus suggest that the genes in this scheme did not have sufficient discrimination and/or typing power for intra-taxa classification. This could have been related to the high degree of genome conservation between *Ct* serovarieties (~99%) (Ferreira et al., 2012), meaning that the use of this scheme should be re-evaluated. These findings were similar to those for Scheme A (also based on housekeeping genes), that showed a low discriminatory power and seem not to be quite useful for evaluating intra-taxa diversity.

Regarding Scheme C (based on hypervariable genes), intra-taxa analysis identified that most genes in this scheme had heterogeneous percentages of identity and amount of variable and informative sites (Supplementary Figure S4), accompanied by reduced diversity indices (except for the CT172 gene) (Figure 3). Regarding population structure, this scheme produced most CCs (*n* = 15) (Figure 4), showing a high degree of intra-taxa diversity, corroborated by the large number of clusters in the allele plot (except for the C172 gene) (Figure 5) and by the high degree of reticulation in the phylogenetic networks (Supplementary Figure S5). Using schemes exclusively including hypervariable genes could thus overestimate diversity in terms of population structure, as has been observed in other pathogens, such as *Candida albicans* (McManus and Coleman, 2014).

Regarding scheme D, the results showed that all genes in such scheme had high identity (Figure 3 and Supplementary Figure S4), few variable sites and heterogeneity concerning the number of informative sites, including totally informative markers, such as CHLAM0895 and CHLAM0898 (Supplementary Figure S4), as well as low nucleotide diversity, thereby agreeing with the conserved nature previously reported for these elements. Concerning population structure, 6 CC were identified (Figure 4), showing moderate intra-taxa diversity, supported by the number of clusters observed in the allele plot. The CHLAMY0895 gene had high discrimination power (8 clusters) (Figure 5), possibly being an indicator of recombination events. However, the discrete reticulation found in the phylogenetic network (Supplementary Figure S5) suggested that intra-taxa diversity may not have been suitably identified as it has been identified by other markers, such as *OmpA*, where it has been observed that true diversity has been masked (Harris et al., 2012). Plasmid loci's informative capability could be related to mobile genetic elements or errors during transduction events enabling the emergence of molecular rearrangements, ultimately affecting bacterial fitness (Sigar et al., 2014). Recent studies have shown that the presence of these plasmids governs chromosomal gene transcription related to the pathogenic effect, thereby being proposed as virulence factors for this species (Zhong, 2017).

In the absence of a “true scheme” that could help us to determine the best scheme for understanding the *Ct* molecular epidemiology. We decided to retrieve the available *Ct* genomes and compare the cgMLST and SNP cgMLST phylogenies with the available MLST schemes (A–D).

**Figure 6:**
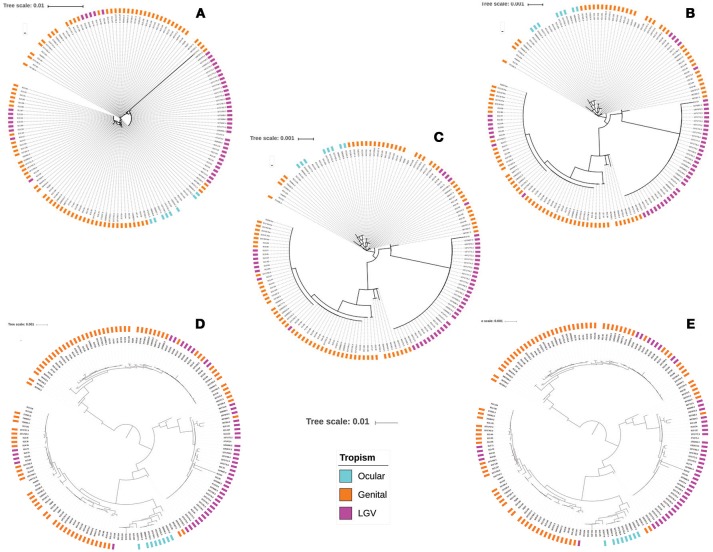
Comparison of phylogenetic reconstructions obtained from genome dataset. Phylogenetic trees after extraction and alignment of seven housekeeping genes included in Chlamydiales MLST schemes: **(A)** Scheme A; **(B)** Scheme B; **(C)** Scheme C; **(D)** Core genome phylogeny. **(E)** SNPs core genome phylogeny.

Initially, the results obtained with the 16S rRNA phylogeny showed the scarce utility of this marker for the intra-species typing of *Ct*, due to its limited discrimination and genotyping power (Supplementary Figure S6) and its restricted intra-species classification capacity (presents limited informative sites), mainly in the classification between organisms with closely related genomes (Cooper and Feil, 2004; Carrasco et al., 2013). Regarding phylogenies inferred using core genome and SNPs-core genome, they showed similar topology and clustering according to tropism. The results also confirm that phylogenetic approaches that start from complete genomes, provide a greater discriminatory power at the intra-species level (Figure 6) (Versteeg et al., 2018); typing schemes aimed at the core genome allow to detect minimum changes at the genome level between variants, allowing a more robust classification. This methodology is advantageous and presents better resolution to those schemes based on MLST, mainly in microorganisms with highly conserved genomes (Gonzalez-Escalona et al., 2017; Tsang et al., 2017; Versteeg et al., 2018). However, in general the clustering (using genomic data) did not significantly differ from that based on MLST schemes herein evaluated. In terms of feasibility, we conclude that MLST might be more accessible and hypothesis driven than cgMLST.

**Figure 7:**
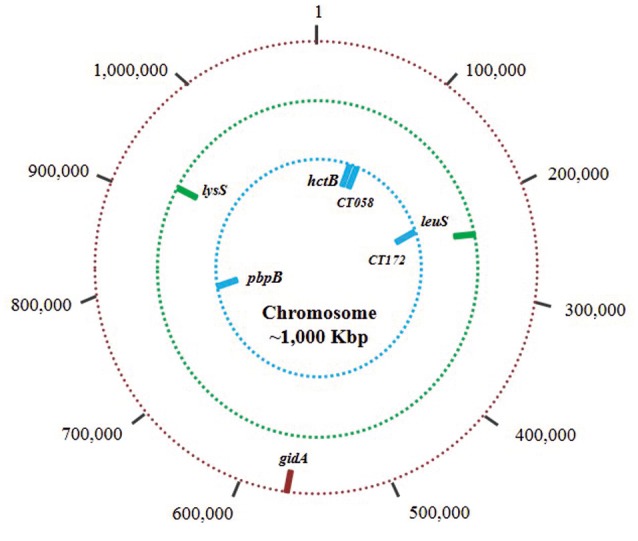
Chromosomal markers suggested as the best combination for *Ct* typing.

Despite the schemes evaluated have been developed for different purposes (schemes A,B have been suitable for evolutionary studies and the scheme C for short-term clinical epidemiology and outbreak investigations) and have been based on different targets (housekeeping and hypervariable genes), which can generate a bias in the analyses obtained. They represent the only information currently available worldwide for genotyping Chlamydiales. The findings show that the four MLST schemes available for *Ct* described to date do not have suitable behavior for describing circulating genotypes and thus cannot adequately describe inter- and intra-taxa diversity. However, analysis of individual markers showed compliance with the criteria required for being used in an MLST scheme, i.e., suitable typing efficiency, high discrimination power and a lack of stabilizing selective pressure (dN/dS lower than 1.0) (Table 1). Analyzing the optimisation of the scheme using the set of markers having the best behavior (Table 2) led to suggesting the use of 3 loci, currently included in B (housekeeping gene), C (hyper-variable gene), and D MLST schemes (plasmid loci), as being the best combination of genes for *Ct* typing (Figure 7). They had optimized typing efficiency concerning the dataset evaluated here and also maintained maximum discrimination power. Even though some isolates lose plasmids (Sigar et al., 2014), their important role regarding the impact of *Ct* on a particular host ratifies their usefulness as typing marker, meaning that even their absence should be considered within a scheme applicable to clinical isolates.

However, in circumstances where access to the core genome is restricted, either by the availability of information, samples or by computational tools (Yi and Jin, 2013; Taylor-Brown et al., 2016), the MLST could be considered as a good tool for intra-species typing because it has been shown to be reproducible, highly discriminatory and easy to implement in the laboratory (Cooper and Feil, 2004). Although its use can lead to analysis of small-scale evolutionary changes, given the use of only one set of molecular markers (Tsang et al., 2017). Herein, we show MLST optimization as an alternative for *Ct* typing that showed to be in overall compatible with the cgMLST and SNP cgMLST. It is well known that the WGS is the best tool to assess the variability and to improve the understanding of inter- and intraspecies phylogenetic relationships (Tsang et al., 2017); however, this requires a more complex infrastructure, as its higher costs and the analysis of the data is more complex. With this article we intend to generate a cost-effective tool that allow an identification of circulating strains in short time, which in the future may contribute to characterizing outbreak transmission, monitoring relapses (recurrence/reinfection) (de Vries et al., 2015), and identifying the genetic variability of species infecting multiple hosts. Taken together, the above will contribute toward the surveillance of emergent genotypes and understanding the genetic causes of the disease's physiopathological mechanisms.

## Author contributions

LP, MC, MM and JR conceived and designed the study, analyzed and interpreted the data and prepared the manuscript. DR-C, JR and MP critically read the manuscript and made important suggestions. JR conceived and designed the study and revised the manuscript. All authors have reviewed and approved the manuscript.

### Conflict of interest statement

The authors declare that the research was conducted in the absence of any commercial or financial relationships that could be construed as a potential conflict of interest.
