# Supplementary material for: Unveiling the Multilocus Sequence Typing (MLST) Schemes and Core Genome Phylogenies for Genotyping Chlamydia trachomatis
Source: Front Microbiol. 2018 Aug 22;9:1854. doi: 10.3389/fmicb.2018.01854 (PMC6113918; doi:10.3389/fmicb.2018.01854)
Supplement: Supplementary file 1 [file Data_Sheet_1.pdf]

Supplementary Material

Unveiling the Multilocus Sequence Typing (MLST) schemes and Core Genome phylogenies for genotyping *Chlamydia trachomatis*

Luz Helena Patiño[1,2†], Milena Camargo [2,3†], Marina Muñoz [1†], Dora Inés Ríos-Chaparro[1], Manuel Alfonso Patarroyo [3,4], Juan David Ramírez [1]*

[1] Universidad del Rosario, Facultad de Ciencias Naturales y Matemáticas, Programa de Biología, Grupo de Investigaciones Microbiológicas UR (GIMUR), Bogotá, Colombia
[2] PhD Programme in Biomedical and Biological Sciences, Universidad del Rosario, Bogotá, Colombia.
[3] Molecular Biology and Immunology Department, Fundación Instituto de Inmunología de Colombia (FIDIC), Bogotá, Colombia
[4] Universidad del Rosario, School of Medicine and Health Sciences, Bogotá, Colombia

[†] The three authors contributed equally as first author

* Corresponding author: Juan David Ramírez BSc, MSc, Dr.Sc.
Address: Carrera 24 # 63C - 69, Bogotá, Colombia
Telephone number: +57-1-297 0200 Ext: 4033
E-mail: juand.ramirez@urosario.edu.co

Supplementary Material

Summary

The supplementary material includes 6 figures and 6 tables.

Supplementary information legends

Supplementary Table S1. Description of Scheme A (Chlamydiales-*Ct*), Scheme B (*C. trachomatis*) and Scheme C (*C. trachomatis* Uppsala) marker location and function analysed here.

Supplementary Figure S1. Ct variant distribution by country.

Supplementary Table S2. Descriptive patterns of interest for Ct.

Supplementary Figure S2. Variant frequency by age group; green shows cluster 1 variants (associated with ocular infections), red shows cluster 2 variants (associated with urogenital infections) and blue cluster 3 variants (associated with Lymphogranuloma venereum). **A.** Isolated from 16 to 29 years-old (n=534) **B.** Isolated from people aged over 29 years-old (n=26).

Supplementary Figure S3. Amount of STs assigned by each MLST scheme and regarding C. trachomatis variants for 3,241 isolates.

Supplementary Table S3. ST distribution frequency regarding to MLST scheme C. trachomatis variant (others include STs having less than 2.0% prevalence).

Supplementary Figure S4. Characteristics of the markers used in the MLST schemes analysed here. **A.** Percentage allele identity per marker. **B.** The number of variant sites per marker compared to the number of informative sites according to parsimony.

Supplementary Table S4. Diversity indexes for the markers in the MLST schemes analysed here. **A.** Scheme A (Chlamydiales-*Ct*). **B.** Scheme B (C. trachomatis). **C.** Scheme C (C. trachomatis Uppsala). **D.** Scheme D (Plasmid loci).

Supplementary Table S5. Description of the STs and clone complexes for the structure observed in the eBURST diagram, according to the scheme being analysed.

Supplementary Figure S5. Concatenated sequence phylogenetic networks for each MLST scheme analysed, based on the Neighbor-net algorithm.

Supplementary Figure S6. Phylogenetic reconstruction based on 16SrRNA from whole genome datataset.

Supplementary Table S6. Analysis of scheme optimisation and optimum number of loci for the 8 molecular markers selected here.

Supplementary Table S1.

Scheme	Locus	Name	Gene start/Stop	Gene length	Function
Chlamydiales-*Ct*	*gatA*	glutamyl-tRNA amidotransferase subunit A	2108-3580*/ 2759-3183[ł]	1473	Allows the formation of correctly charged Gln-tRNA(Gln) through the transamidation of misacylated Glu-tRNA(Gln) in organisms which lack glutaminyl-tRNA synthetase.
	oppA	Oligopeptide Binding Protein	157100-155823*/ 224745-225217 [ł]	1278	Transport and binding; amino acids, peptides and amines
	hflX	GTP-binding protein	433641-432301*/ 435569-435135 [ł]	1341	GTPase that associates with the 50S ribosomal subunit and may have a role during protein synthesis or ribosome biogenesis.
	gidA	tRNA (racil-5-)-methyltransferase	576941-578770*/ 580102-580575 [ł]	1830	NAD-binding protein involved in the addition of a carboxymethylaminomethyl (cmnm) group at the wobble position (U34) of certain tRNAs, forming tRNA-cmnm5s2U34.
	enoA	Enolase	661850-663121*/ 665021-66540 [ł]	1272	Catalyzes the reversible conversion of 2-phosphoglycerate into phosphoenolpyruvate. It is essential for the degradation of carbohydrates via glycolysis.
	hemN	Coproporphyrinogen-III oxidase	60110-58977*/ 871011-870580 [ł]	1134	Is involved in step 1 of the subpathway that synthesizes protoporphyrinogen-IX from coproporphyrinogen-III (AdoMet route).
	fumC	Fumarate hydratase	1004550-1005938*/ 1007488-1007952 [ł]	1389	Catalyzes the reversible addition of water to fumarate to give L-malate.
C.trachomatis	*glyA*	Serine hydroxymethyltransferase	502443/500953	1491	Catalyzes the reversible interconversion of serine and glycine with tetrahydrofolate (THF) serving as the one-carbon carrier.
	mdhC	malate dehydrogenase	430162/429185	978	Catalyzes the reversible oxidation of malate to oxaloacetate.
	pdhA	pyruvate dehydrogenase	273563/274582	1020	Catalyzes the overall conversion of pyruvate to acetyl-CoA and CO2.

	yhbG	ABC transporter ATP binding protein	751062/750346	717	Transport and binding. Membrane Transport; ATP-binding cassette (ABC) transport system.
	pykF	pyruvate kinase	374217/375671	1455	This protein is involved in step **5** of the subpathway that synthesizes pyruvate from D-glyceraldehyde 3-phosphate
	lysS	lysyl-tRNA synthetase	916214/917791	1578	Translation; aminoacyl-tRNA synthetases. Amino Acid Metabolism; Lysine biosynthesis<br>Metabolism of Macromolecules; Aminoacyl-tRNA biosynthesis
	leuS	leucyl-tRNA synthetase	238225/235769	2457	Translation; aminoacyl-tRNA synthetase. Metabolism of Macromolecules; Aminoacyl-tRNA biosynthesis
C.t Uppsala	*hctB*	Histone H1-like protein HC2	51504/52112	609	Might have a role in establishing the nucleoid structure of elementary bodies.
C.t Uppsala	*pbpB*	Penicillin binding protein	780668/783907	3240	Code for a penicillin-binding protein
C.t Uppsala	CT058				Putative uncharacterized protein
C.t Uppsala	CT144				Putative uncharacterized protein
C.t Uppsala	CT172				Putative uncharacterized protein

*Position in genome of Ct A/ HAR-13. Pannekoek, Y. et al. Multi locus sequence typing of Chlamydiales: clonal groupings within the obligate intracellular bacteria Chlamydia trachomatis. BMC microbiology 8, 42, doi:10.1186/1471-2180-8-42 (2008).

[1]Pannekoek Y, et al., Multi locus sequence typing of Chlamydia reveals an association between Chlamydia psittaci genotypes and host species. PLoS One 5, 12:e14179, doi: 10.1371/journal.pone.0014179.2010 (2010)

Supplementary Figure S1.

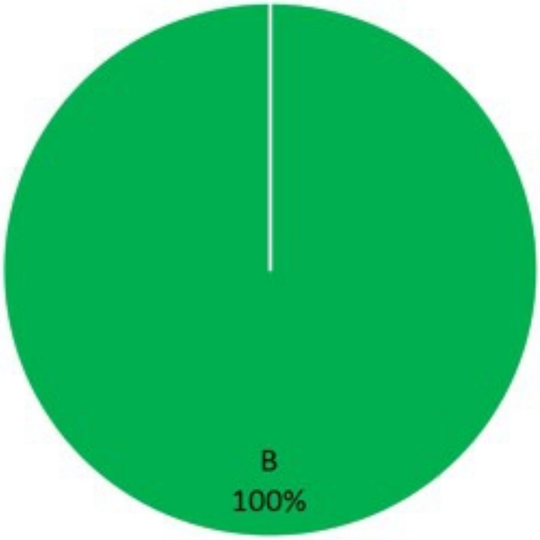

Gambia (n= 1)

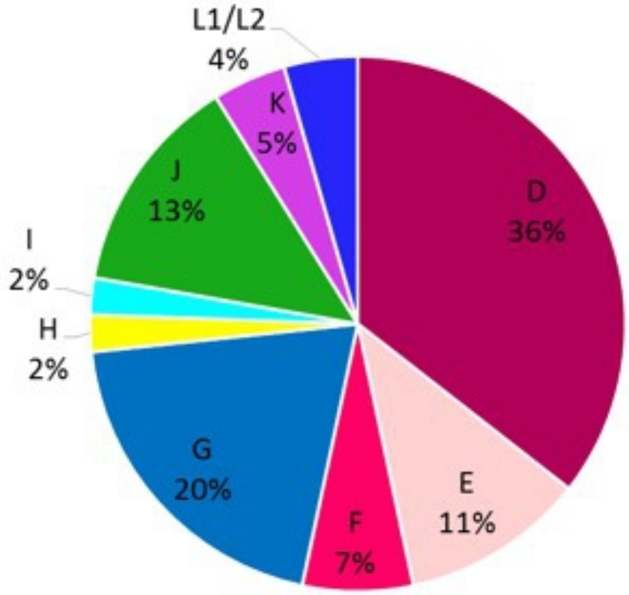

South Africa (n= 45)

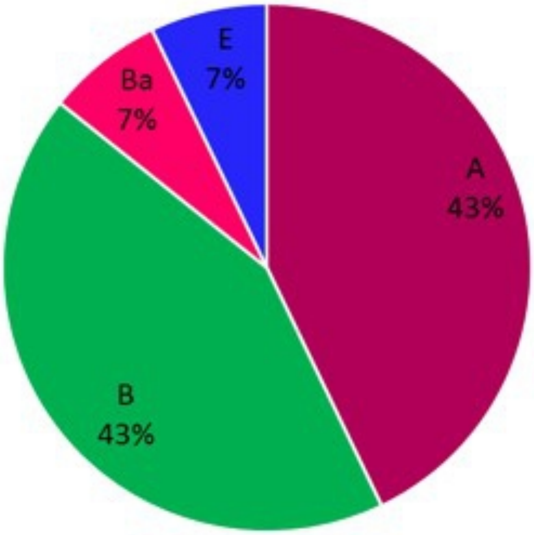

Tanzania (n= 14)

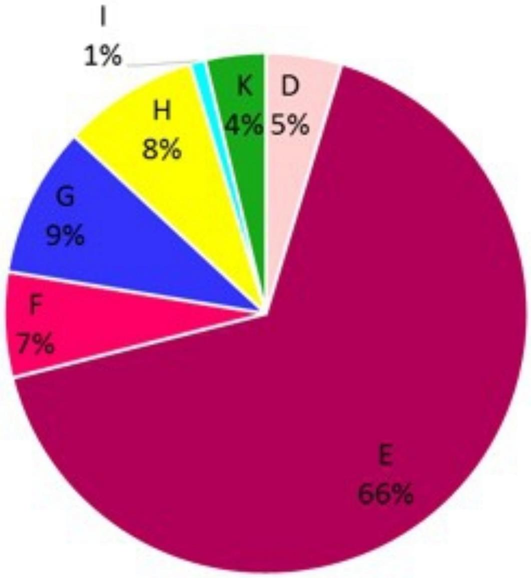

Tunisia (n= 107)

Africa

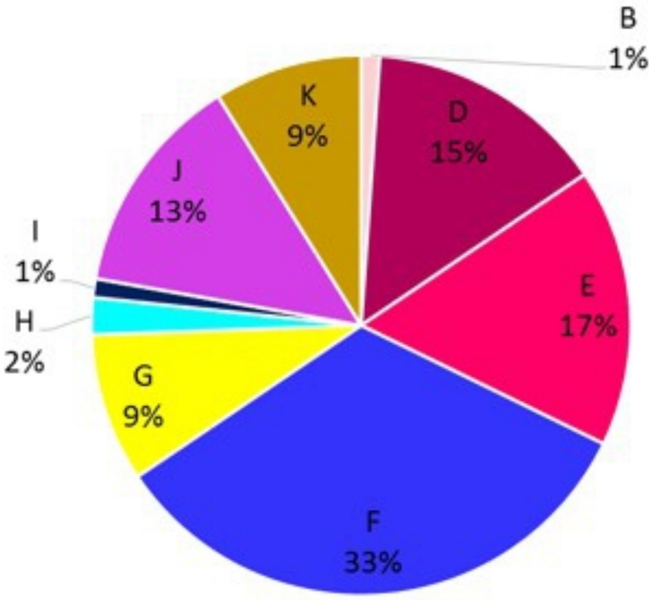

China (n= 90)

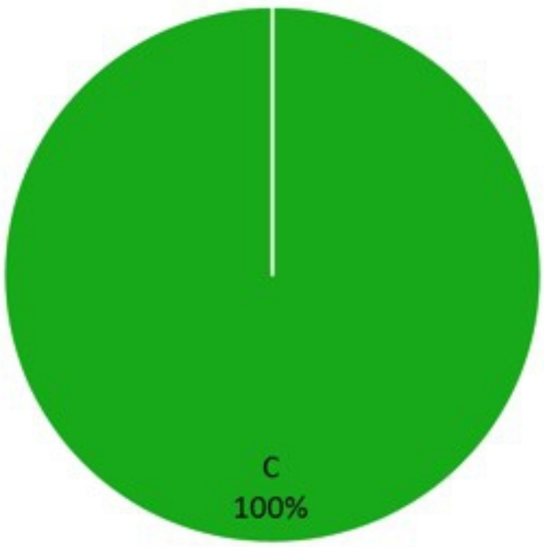

Nepal (n= 10)

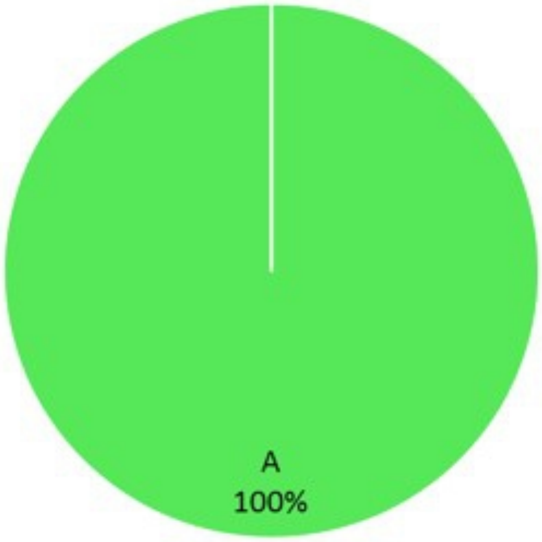

Saudi Arabia (n= 2)

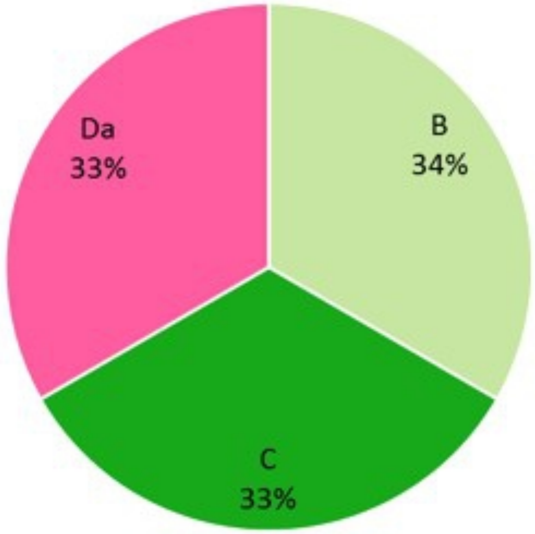

Taiwan (n= 3)

Asia

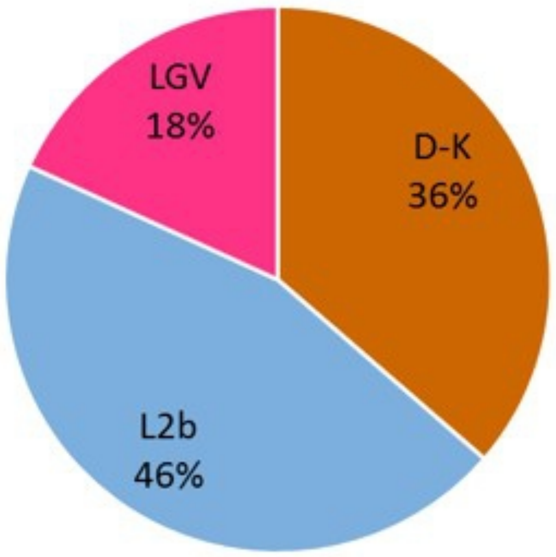

Denmark (n= 11)

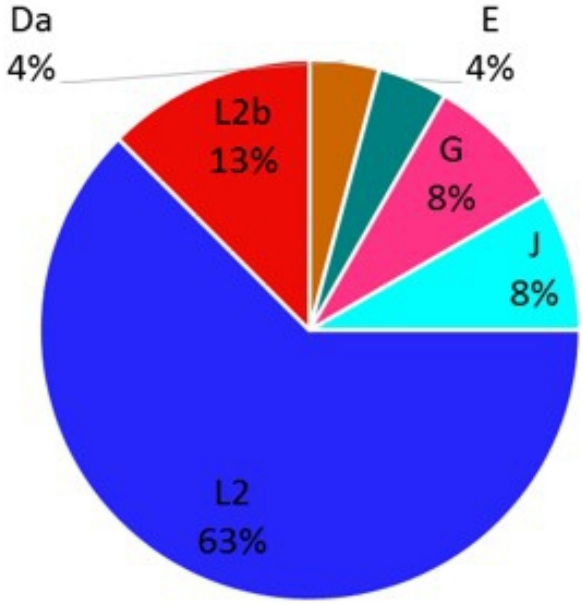

France (n= 24)

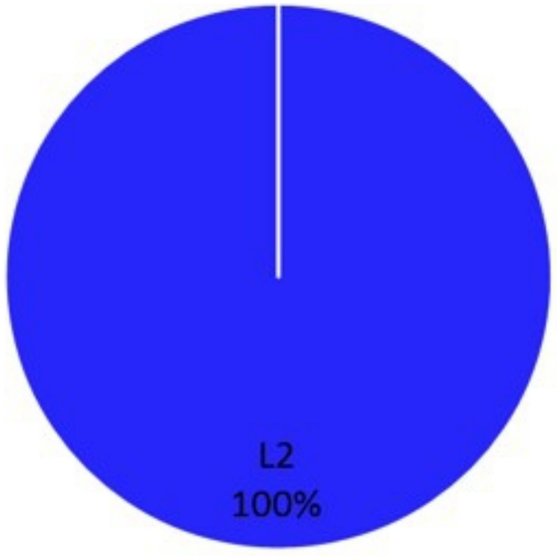

Germany (n= 1)

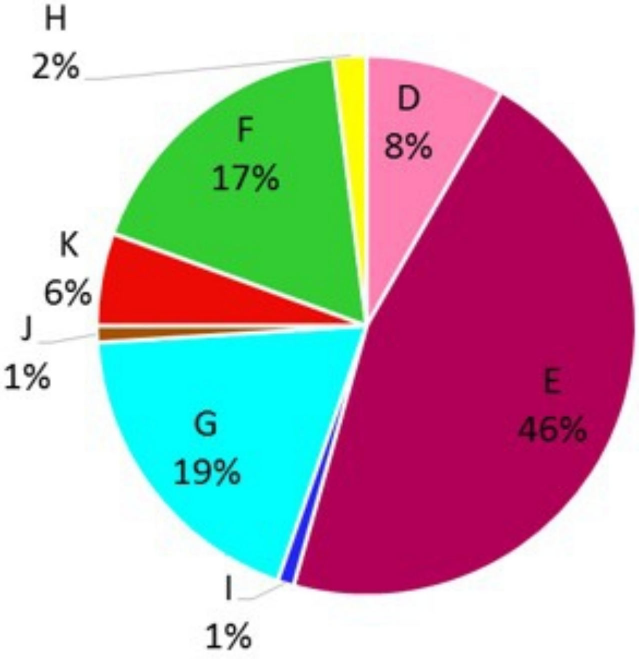

Norway (n= 249)

Europe

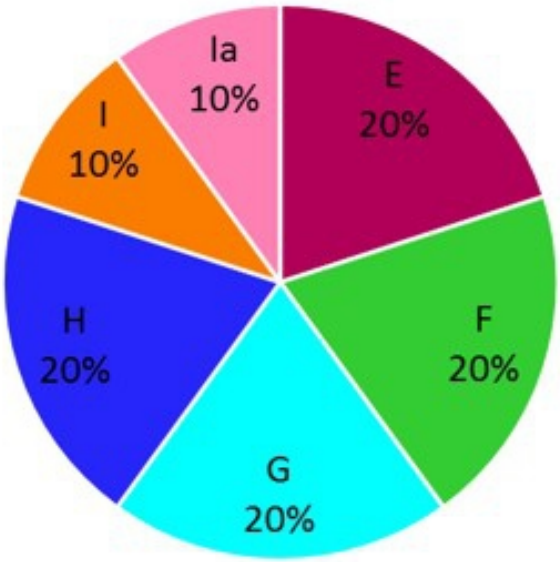

Portugal (n= 10)

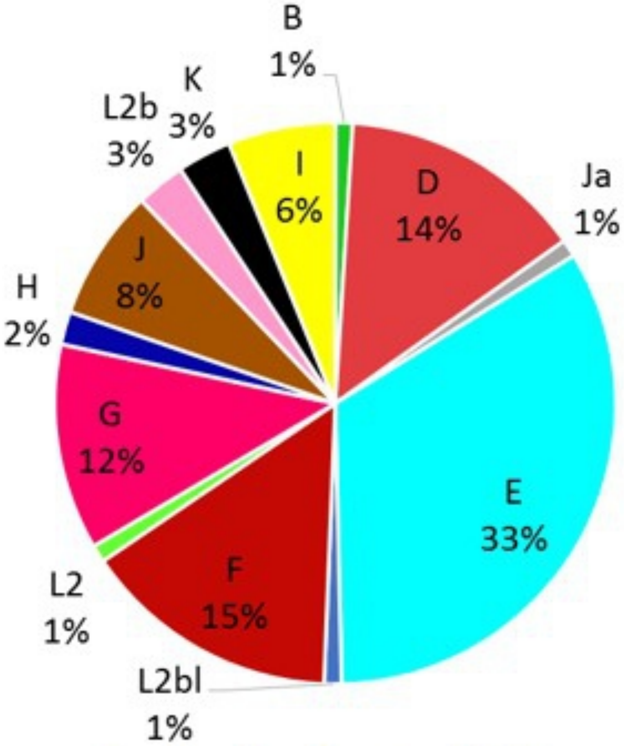

Netherlands (n= 1793)

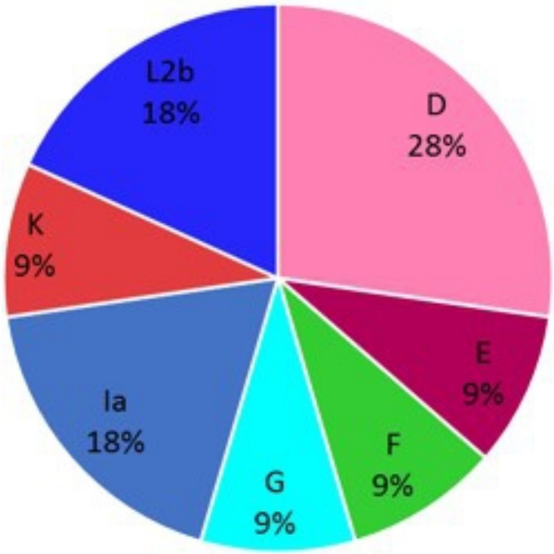

United Kingdom (n= 11)

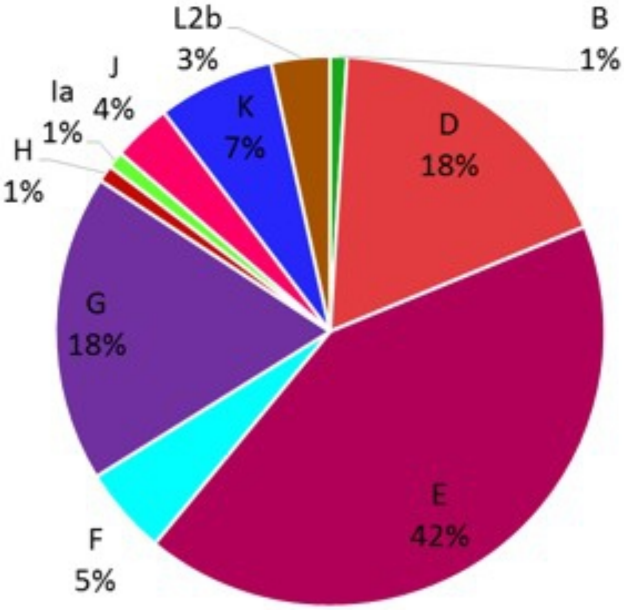

Sweden(n= 342)

Europe

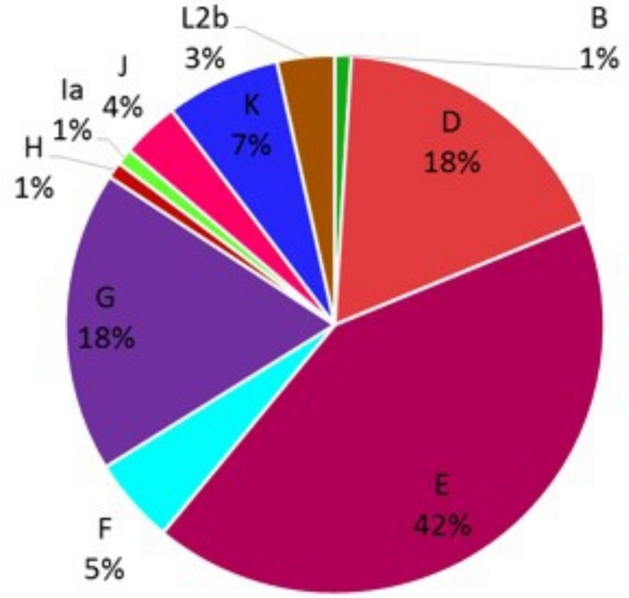

Sweden (n= 342)

Europe

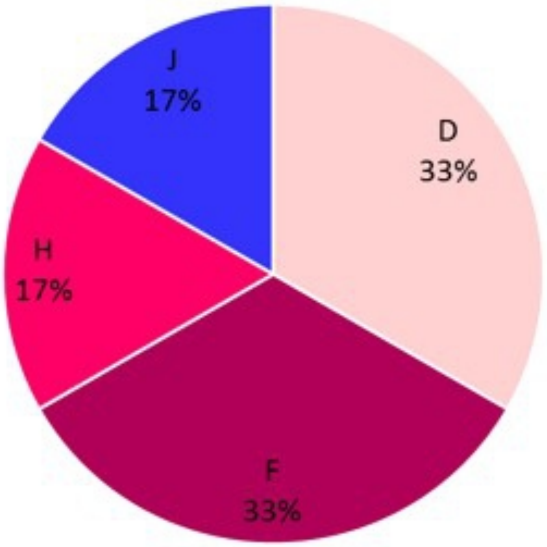

Australia (n= 6)

Oceania

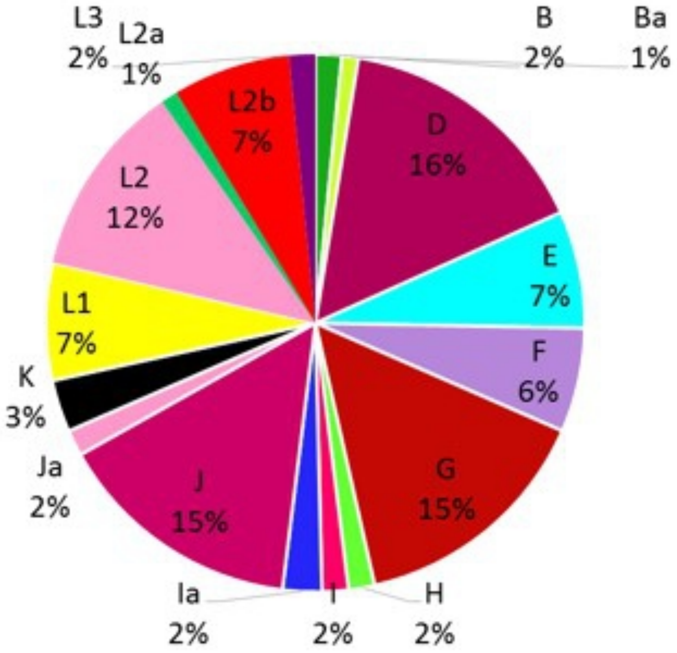

United States (n= 127)

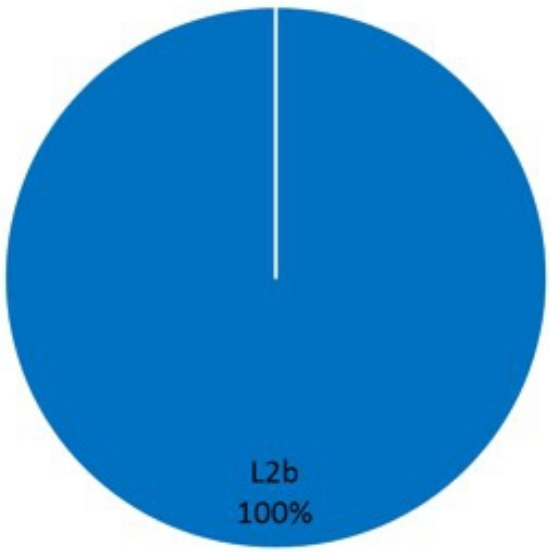

Canada (n= 2)

North America

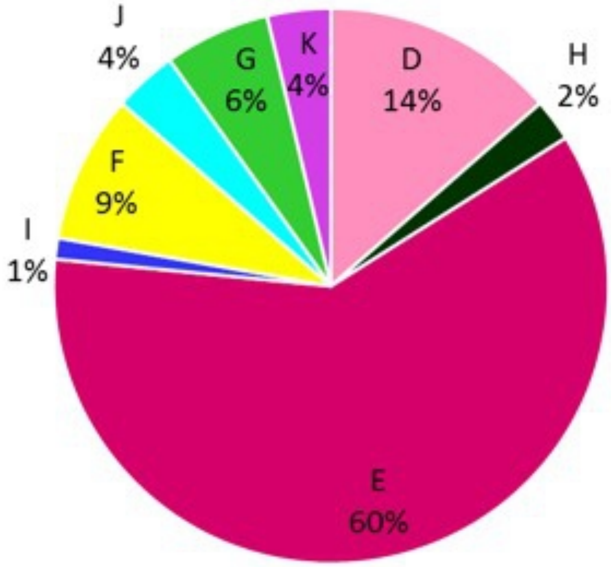

Argentina (n= 81)

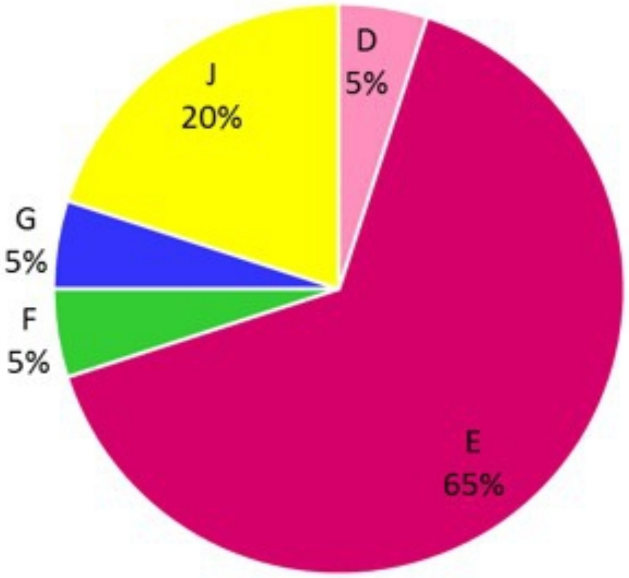

Chile (n= 20)

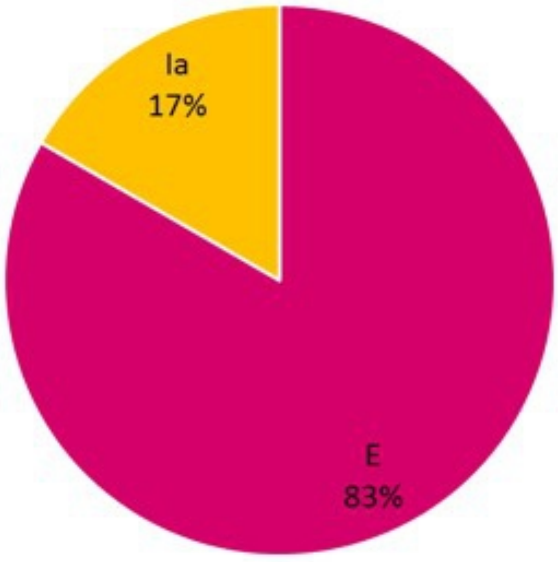

Ecuador (n= 6)

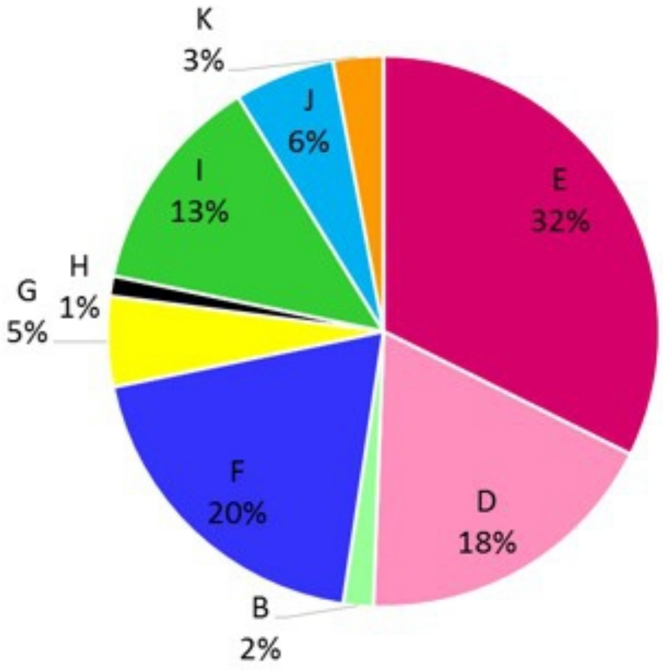

Suriname (n= 170)

South America

Supplementary Table S2.

<table>
<tr><th>Characteristic</th><th>Number of DS isolates with information</th><th colspan="2">Description</th></tr>
<tr><td>Gender</td><td>3,129</td><td colspan="2">50.3% (48.5-52.1 95%CI) of the samples in the database corresponded to men.</td></tr>
<tr><td>Sample source (Fig 3C)</td><td>2,194</td><td colspan="2">The samples with the highest and lowest number of reports were genitourinary tract and pharynx, respectively. Cluster 1 (Variant B) was obtained exclusively from the genitourinary tract. Cluster 3 (Variants L2 and L2b) was obtained from the anal region</td></tr>
<tr><td>Ct distribution (Fig 3B)</td><td>3,133</td><td colspan="2">57.2% de Ct were reported in the Netherlands</td></tr>
<tr><td>Cluster distribution (Supplementary Figure S1)</td><td>3,225</td><td colspan="2">Variants included in Cluster 2 had the highest frequency regarding all regions evaluated
Variants included in Cluster 1 was more prevalent in Asia
Variants included in Cluster 3 was more prevalent in North America</td></tr>
<tr><td rowspan="2">Variant distribution (Fig 3A)</td><td rowspan="2">3,692</td><td>The cluster having the highest report frequency was the Cluster 2: Urogenital infections (D-K, Da, Ga, Ia and Ja) (91.3%: 90.2-92.2 95%CI).</td><td>Variant E (33.7%: 32.1-35.4 95%CI)
Variant D (14.2%: 13.0-15.4 95%CI)
Variant F (13.6%: 12.4-14.8 95%CI)
Variant G (12.5%: 11.4-13.7 95%CI)</td></tr>
<tr><td colspan="2">Cluster 1: Ocular infections and Cluster 3: Lymphogranuloma venereum, having less than 6% prevalence.</td></tr>
<tr><td rowspan="3">Age (Supplementary Figure S2)</td><td rowspan="3">567</td><td>7 isolates obtained from children aged less than 12 years-old (1.2%: 0.04-0.25 95%CI)</td><td>Only the Variant C (Cluster 1) was reported in this group</td></tr>
<tr><td>534 isolates from people aged 16-29 years-old (94.2%: 91.9-95.9 95%CI)</td><td rowspan="2">The Variants E (37.2%: 33.0-41.4 95%CI) and D (34.6%: 17.2-55.6 95%CI) (Cluster 2) were identified in this categories</td></tr>
<tr><td>26 isolates from adults over 29 years of age (4.6%: 3.0-6.6 95%CI)</td></tr>
</table>

Supplementary Figure S2.

16 - 29 years-old

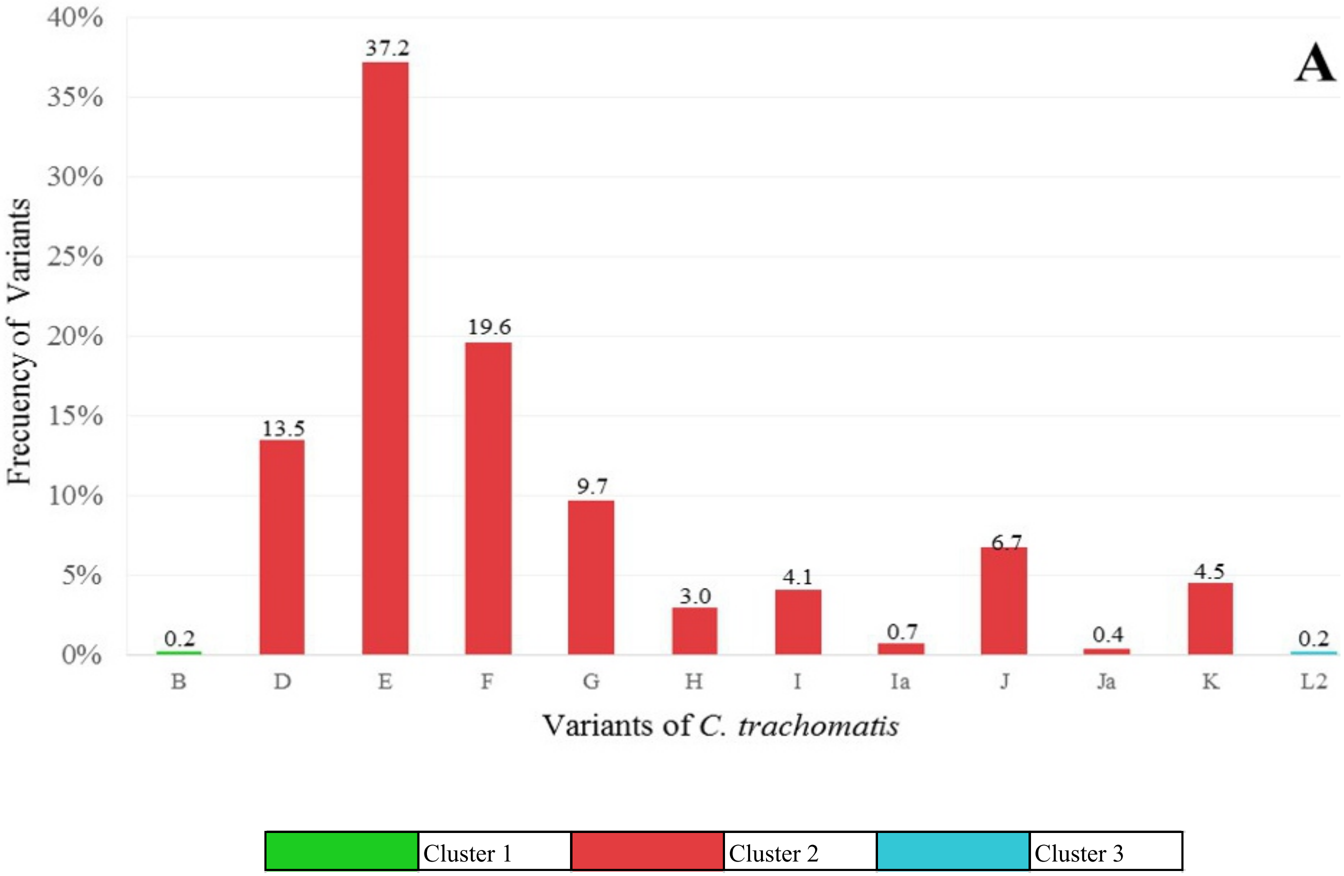

> 29 years-old

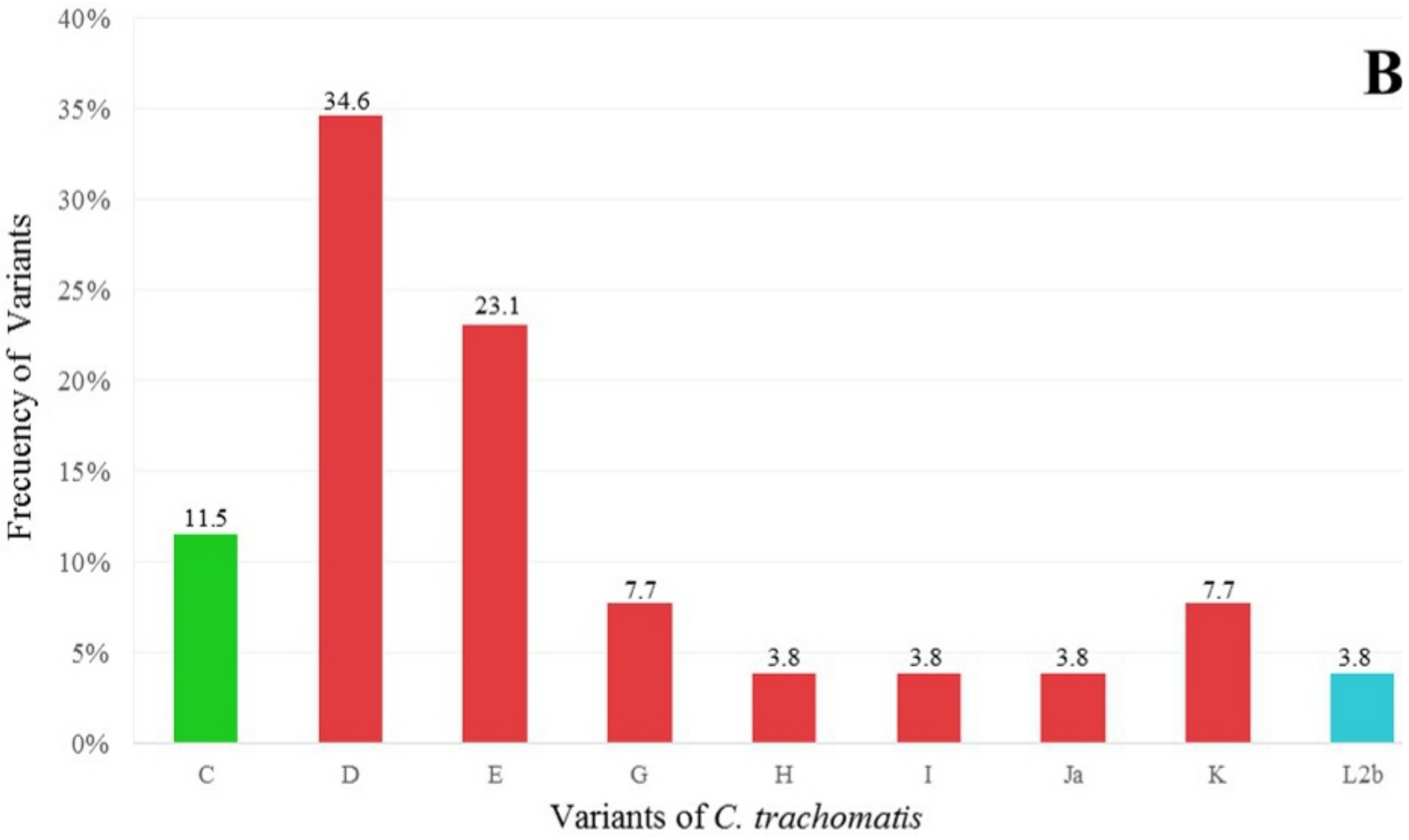

Supplementary Figure S3.

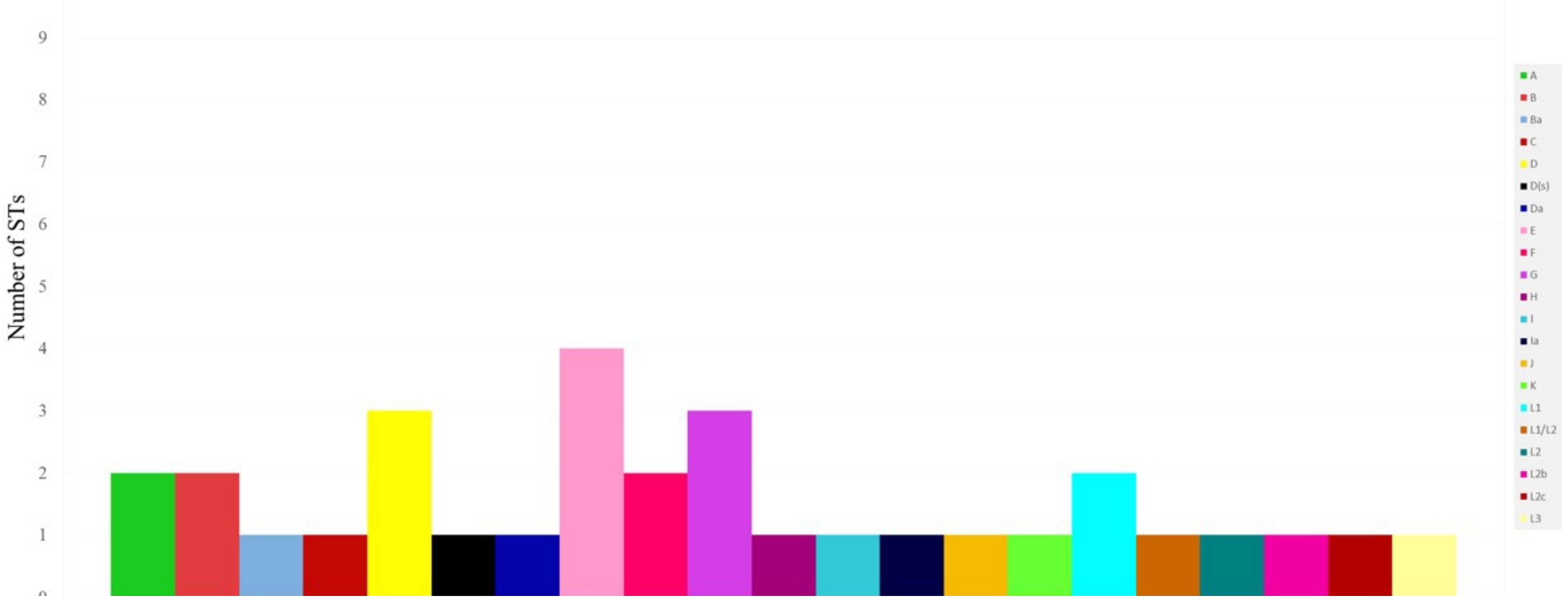

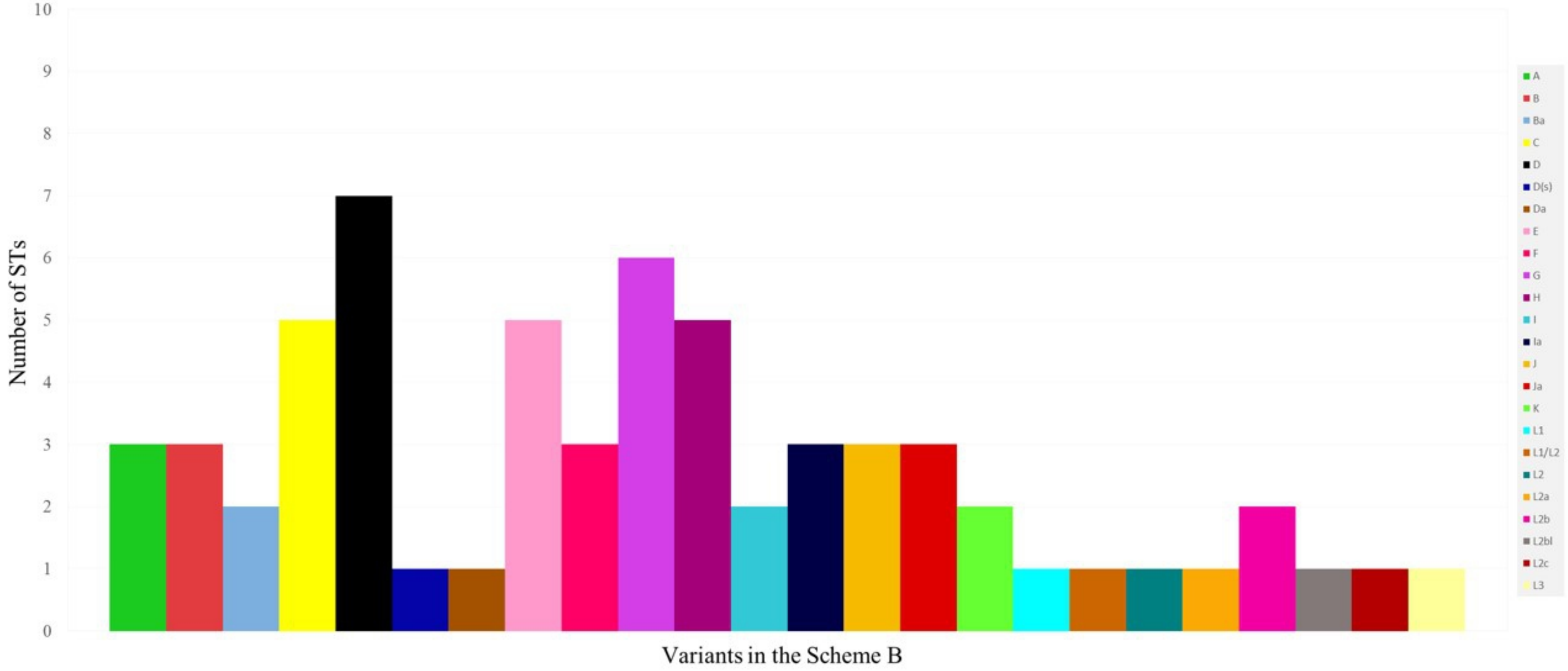

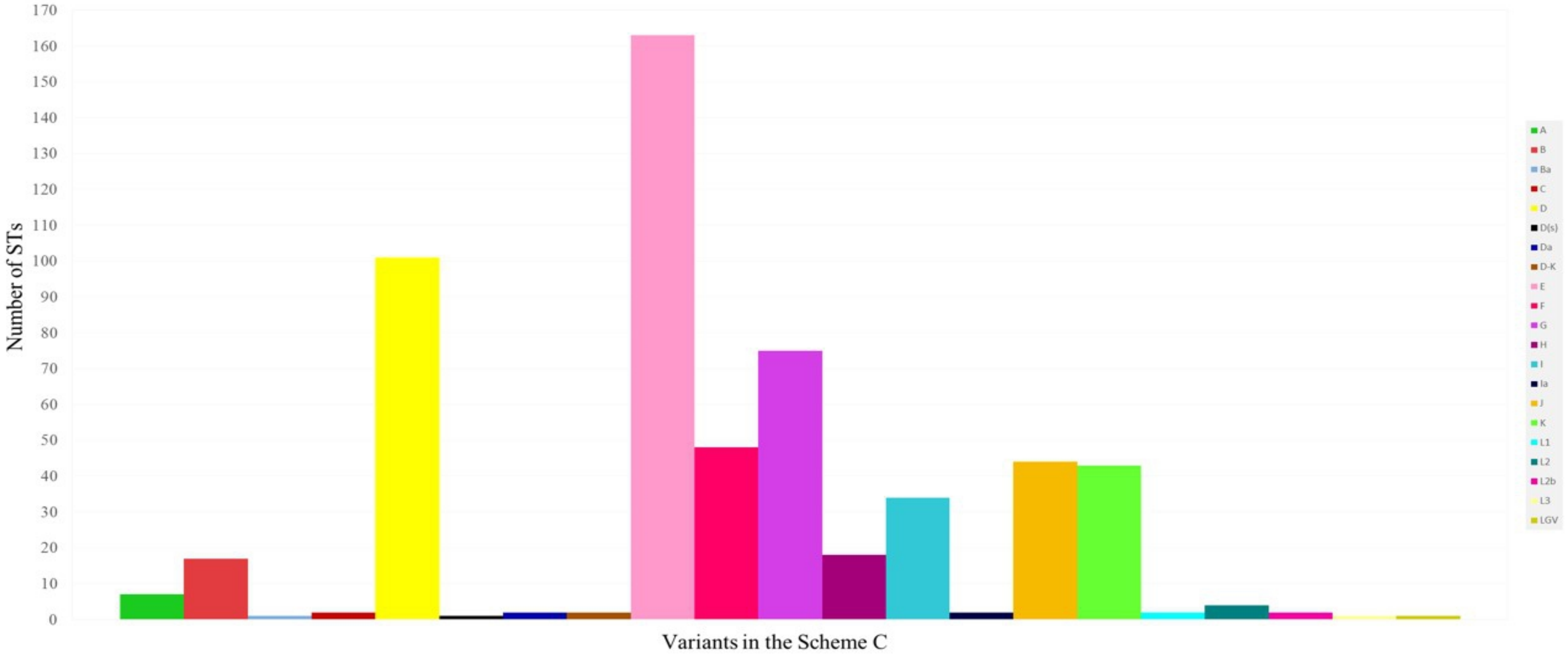
170
160
150
140
130
120
110
100
90
80
70
60
50
40
30
20
10
0
Number of STs
Variants in the Scheme C
A
B
Ba
C
D
D(s)
Da
D-K
E
F
G
H
I
Ia
J
K
L1
L2
L2b
L3
LGV

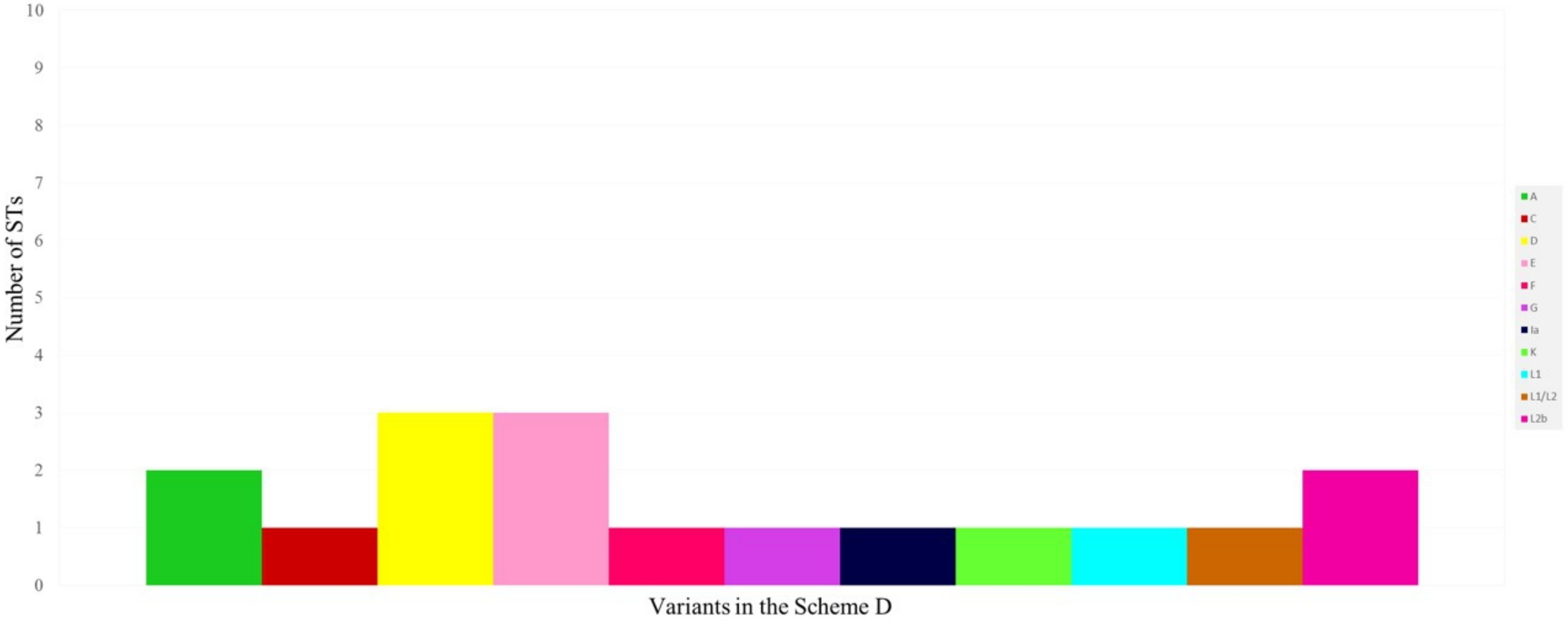
10
9
8
7
6
5
4
3
2
1
0
Number of STs
Variants in the Scheme D
A
C
D
E
F
G
Ia
K
L1
L1/L2
L2b

Supplementary Table S3.

MLST	Scheme A			Scheme B			Scheme C			Scheme D		
Variant	*ST*	Isolate (n)	%	*ST*	Isolate (n)	%	*ST*	Isolate (n)	%	*ST*	Isolate (n)	%
A	*3*	6	85.7	*4*	7	77.8	*119*	28	43.1	*4*	4	80.0
n= 75	*1*	1	14.3	*12*	1	11.1	*115*	20	30.8	*1*	1	20.0
				22	1	11.1	*47*	8	12.3			
							118	6	9.2			
							Others	3	4.6			
**Total**	***2***	**7**		***3***	**9**		***7***	**65**		***2***	**5**	

MLST	Scheme A			Scheme B			Scheme C			Scheme D		
Variant	*ST*	Isolate (n)	%	*ST*	Isolate (n)	%	*ST*	Isolate (n)	%	*ST*	Isolate (n)	%
B	*3*	1	50.0	*12*	5	71.4	*118*	4	13.3			
n= 38	*45*	1	50.0	*6*	1	14.3	*138*	4	13.3			
				10	1	14.3	*74*	3	10.0			
							120	3	10.0			
							124	2	6.7			
							211	2	6.7			
							479	2	6.7			
							48	1	3.3			
							123	1	3.3			
							125	1	3.3			
							126	1	3.3			
							208	1	3.3			
							259	1	3.3			
							280	1	3.3			
							401	1	3.3			
							487	1	3.3			
							488	1	3.3			
**Total**	***2***	**2**		***3***	**7**		***17***	**30**		***0***	**0**	

MLST	Scheme A			Scheme B			Scheme C			Scheme D		
Variant	*ST*	Isolate (n)	%	*ST*	Isolate (n)	%	*ST*	Isolate (n)	%	*ST*	Isolate (n)	%
Ba	*3*	1	100	*12*	1	50.0	*4*	1	100			
n= 2				*18*	1	50.0						
**Total**	***1***	**1**		***2***	**2**		***1***	**1**		***0***	**0**	

MLST	Scheme A			Scheme B			Scheme C			Scheme D		
Variant	*ST*	Isolate (n)	%	*ST*	Isolate (n)	%	*ST*	Isolate (n)	%	*ST*	Isolate (n)	%
C	*3*	1	100	*44*	6	54.5	*46*	1	50.0	*33*	1	100
n= 12				*13*	2	18.2	*177*	1	50.0			
				11	1	9.1						
				42	1	9.1						
				43	1	9.1						
**Total**	***1***	**1**		***5***	**11**		***2***	**2**		***1***	**1**	

MLST	Scheme A			Scheme B			Scheme C			Scheme D		
Variant	*ST*	Isolate (n)	%	*ST*	Isolate (n)	%	*ST*	Isolate (n)	%	*ST*	Isolate (n)	%
D	*13*	6	75.0	*19*	3	33.3	*109*	113	24.9	*6*	2	40.0
n= 461	*4*	1	12.5	*2*	1	11.1	*12*	62	13.7	*9*	2	40.0
	38	1	12.5	*20*	1	11.1	*35*	39	8.6	*8*	1	40.0
				23	1	11.1	*77*	19	4.2			
				25	1	11.1	*11*	11	2.4			
				34	1	11.1	*14*	10	2.2			
				38	1	11.1	*20*	10	2.2			
							13	9	2.0			
							85	9	2.0			
							248	9	2.0			
							395	9	2.0			
							Others	154	33.9			
**Total**	***3***	**8**		***7***	**9**		***101***	**454**		***3***	**5**	

MLST	Scheme A			Scheme B			Scheme C			Scheme D		
Variant	*ST*	Isolate (n)	%	*ST*	Isolate (n)	%	*ST*	Isolate (n)	%	*ST*	Isolate (n)	%
D(s)	*38*	1	100	***34***	1	100	***12***	1	100			
n= 1												
**Total**	***1***	**1**		***1***	**1**		***1***	**1**		***0***	**0**	

MLST	Scheme A			Scheme B			Scheme C			Scheme D		
Variant	*ST*	Isolate (n)	%	*ST*	Isolate (n)	%	*ST*	Isolate (n)	%	*ST*	Isolate (n)	%
Da	*5*	1	100	*37*	1	100	*85*	1	50.0			
n= 3							*109*	1	50.0			
**Total**	***1***	**1**		***1***	**1**		***2***	**2**		***0***	**0**	

MLST	Scheme A			Scheme B			Scheme C			Scheme D		
Variant	*ST*	Isolate (n)	%	*ST*	Isolate (n)	%	*ST*	Isolate (n)	%	*ST*	Isolate (n)	%
D-K							*109*	3	75.0			
n= 4							*56*	1	25.0			
**Total**	***0***	**0**		***0***	**0**		***2***	**4**		***0***	**0**	

MLST	Scheme A			Scheme B			Scheme C			Scheme D		
Variant	*ST*	Isolate (n)	%	*ST*	Isolate (n)	%	*ST*	Isolate (n)	%	*ST*	Isolate (n)	%
E	*4*	6	66.7	*39*	15	75.0	*56*	219	20,3	*2*	1	33.3
n= 1095	*8*	1	11.1	*34*	2	10.0	*3*	212	19.6	*12*	1	33.3
	12	1	11.1	*17*	1	5.0	*55*	86	8.0	*13*	1	33.3
	94	1	11.1	*36*	1	5.0	*59*	63	5.8			
				40	1	5.0	*153*	42	3.9			
							16	32	3.0			
							69	32	3.0			
							64	22	2.0			
							Others	373	34.5			
**Total**	***4***	**9**		***5***	**20**		***163***	**1081**		***3***	**3**	

MLST	Scheme A			Scheme B			Scheme C			Scheme D		
Variant	*ST*	Isolate (n)	%	*ST*	Isolate (n)	%	*ST*	Isolate (n)	%	*ST*	Isolate (n)	%
F	*38*	3	75.0	*34*	6	60.0	*12*	231	53.1	*8*	1	100
n= 442	*7*	1	25.0	*35*	3	30.0	*110*	33	7.6			
				32	1	10.0	*148*	28	6.4			
							90	26	6.0			
							13	16	3.7			
							91	16	3.7			
							62	15	3.4			
							Others	70	16.1			
**Total**	***2***	**4**		***3***	**10**		***48***	**435**		***1***	**1**	

MLST	Scheme A			Scheme B			Scheme C			Scheme D		
		Isolate			Isolate			Isolate			Isolate	
Variant	*ST*	(n)	%	*ST*	(n)	%	*ST*	(n)	%	*ST*	(n)	%
G	*9*	3	60.0	*21*	2	28.6	*52*	103	25.8	*9*	1	100
N= 407	*6*	1	20.0	*14*	1	14.3	*33*	44	11.0			
	95	1	20.0	*19*	1	14.3	*27*	24	6.0			
				26	1	14.3	*137*	24	6.0			
				27	1	14.3	*128*	23	5.8			
				30	1	14.3	*161*	16	4.0			
							108	13	3.3			
							94	11	2.8			
							265	11	2.8			
							54	131	32.7			
**Total**	***3***	**5**		***6***	**7**		***75***	**400**		***1***	**1**	

MLST	Scheme A			Scheme B			Scheme C			Scheme D		
		Isolate			Isolate			Isolate			Isolate	
Variant	*ST*	(n)	%	*ST*	(n)	%	*ST*	(n)	%	*ST*	(n)	%
H	*10*	1	100	*19*	2	33.3	*97*	15	26.8			
N= 62				*3*	1	16.7	*165*	8	14.3			
				5	1	16.7	*34*	5	8.9			
				29	1	16.7	*444*	5	8.9			
				31	1	16.7	*220*	3	5.4			
							380	3	5.4			
							442	3	5.4			
							96	2	3.6			
							494	2	3.6			
							544	2	3.6			
							Others	8	14.3			
**Total**	***1***	**1**		***5***	**6**		***18***	**56**		***0***	**0**	

MLST	Scheme A			Scheme B			Scheme C			Scheme D		
Variant	***ST***	**Isolate (n)**	**%**	***ST***	**Isolate (n)**	**%**	***ST***	**Isolate (n)**	**%**	***ST***	**Isolate (n)**	**%**
I	*5*	1	100	*7*	1	50.0	*100*	29	20.9			
n= 141				*9*	1	50.0	*135*	21	15.1			
							101	13	9.4			
							276	13	9.4			
							136	7	5.0			
							274	6	4.3			
							25	5	3.6			
							272	5	3.6			
							518	5	3.6			
							103	3	2.2			
							Others	32	23.0			
**Total**	***1***	**1**		***2***	**2**		***34***	**139**		***0***	**0**	

MLST	Scheme A			Scheme B			Scheme C			Scheme D		
Variant	***ST***	**Isolate (n)**	**%**	***ST***	**Isolate (n)**	**%**	***ST***	**Isolate (n)**	**%**	***ST***	**Isolate (n)**	**%**
Ia	*9*	4	100	*23*	6	66.7	*37*	1	50.0	*16*	4	100
n= 11				*24*	2	22.2	*136*	1	50.0			
				28	1	11.1						
**Total**	***1***	**4**		***3***	**9**		***2***	**2**		***1***	**4**	

MLST	Scheme A			Scheme B			Scheme C			Scheme D		
	ST	**n**	***%***	***ST***	**n**	***%***	***ST***	**n**	***%***	***ST***	**n**	***%***
Variant	***ST***	**Isolate (n)**	***%***	***ST***	**Isolate (n)**	***%***	***ST***	**Isolate (n)**	***%***	***ST***	**Isolate (n)**	***%***
J	*9*	1	100	*9*	1	33.3	*108*	72	35.3			
n= 207				*16*	1	33.3	*264*	15	7.4			
				19	1	33.3	*135*	11	5.4			
							233	9	4.4			
							136	8	3.9			
							112	7	3.4			
							232	7	3.4			
							281	7	3.4			
							12	6	2.9			
							98	6	2.9			
							295	5	2.5			
							530	5	2.5			
							267	4	2.0			
							Others	42	20.6			
**Total**	***1***	**1**		***3***	**3**		***44***	**204**		***0***	**0**	

MLST	Scheme A			Scheme B			Scheme C			Scheme D		
Variant	***ST***	**Isolate (n)**	***%***	***ST***	**Isolate (n)**	***%***	***ST***	**Isolate (n)**	***%***	***ST***	**Isolate (n)**	***%***
Ja				*34*	2	50.0						
n= 4				*9*	1	25.0						
				41	1	25.0						
**Total**	***0***	**0**		***3***	**4**		***0***	**0**		***0***	**0**	

MLST	Scheme A			Scheme B			Scheme C			Scheme D		
Variant	***ST***	**Isolate (n)**	*%*	***ST***	**Isolate (n)**	*%*	***ST***	**Isolate (n)**	*%*	***ST***	**Isolate (n)**	*%*
K	*6*	1	100	*15*	2	66.7	*30*	26	22.0	*9*	2	100
n= 122				*8*	1	33.3	*32*	12	10.2			
							220	10	8.5			
							133	8	6.8			
							34	7	5.9			
							175	7	5.9			
							411	5	4.2			
							168	3	2.5			
							Others	40	33.9			
**Total**	***1***	**1**		***2***	**3**		***43***	**118**		***1***	**2**	

MLST	Scheme A			Scheme B			Scheme C			Scheme D		
Variant	***ST***	**Isolate (n)**	*%*	***ST***	**Isolate (n)**	*%*	***ST***	**Isolate (n)**	*%*	***ST***	**Isolate (n)**	*%*
L1	*11*	2	66.7	*1*	3	100	*142*	7	77.8	*3*	1	100
n= 11	*44*	1	33.3				*49*	2	22.2			
**Total**	***2***	**3**		***1***	**3**		***2***	**9**		***1***	**1**	

MLST	Scheme A			Scheme B			Scheme C			Scheme D		
Variant	***ST***	**Isolate (n)**	*%*	***ST***	**Isolate (n)**	*%*	***ST***	**Isolate (n)**	*%*	***ST***	**Isolate (n)**	*%*
L1/L2	*44*	2	100	*1*	2	100				*19*	2	100
n= 2												
**Total**	***1***	**2**		***1***	**2**		***0***	**0**		***1***	**2**	

MLST	Scheme A			Scheme B			Scheme C			Scheme D		
Variant	***ST***	**Isolate (n)**	*%*	***ST***	**Isolate (n)**	*%*	***ST***	**Isolate (n)**	*%*	***ST***	**Isolate (n)**	*%*
L2	*44*	4	100	*1*	5	100	*58*	21	52.5			
n= 42							*141*	12	30.0			
							143	5	12.5			
							144	2	5.0			
**Total**	***1***	**4**		***1***	**5**		***4***	**40**		***0***	**0**	

MLST	Scheme A			Scheme B			Scheme C			Scheme D		
Variant	*ST*	Isolate (n)	%	*ST*	Isolate (n)	%	*ST*	Isolate (n)	%	*ST*	Isolate (n)	%
L2a				*1*	1	100						
n= 1												
**Total**	***0***	**0**		***1***	**1**		***0***	**0**		***0***	**0**	

MLST	Scheme A			Scheme B			Scheme C			Scheme D		
Variant	*ST*	Isolate (n)	%	*ST*	Isolate (n)	%	*ST*	Isolate (n)	%	*ST*	Isolate (n)	%
L2b	*44*	14	100	*1*	14	87.5	*58*	87	97.8	*21*	11	91.7
n= 92				*33*	2	12.5	*143*	2	2.2	*31*	1	8.3
**Total**	***1***	**14**		***2***	**16**		***2***	**89**		***2***	**12**	

MLST	Scheme A			Scheme B			Scheme C			Scheme D		
Variant	*ST*	Isolate (n)	%	*ST*	Isolate (n)	%	*ST*	Isolate (n)	%	*ST*	Isolate (n)	%
L2bl				*1*	1	100						
n= 1												
**Total**	***0***	**0**		***1***	**1**		***0***	**0**		***0***	**0**	

MLST	Scheme A			Scheme B			Scheme C			Scheme D		
Variant	*ST*	Isolate (n)	%	*ST*	Isolate (n)	%	*ST*	Isolate (n)	%	*ST*	Isolate (n)	%
L2c	*44*	1	100	*1*	1	100						
n= 1												
**Total**	***1***	**1**		***1***	**1**		***0***	**0**		***0***	**0**	

MLST	Scheme A			Scheme B			Scheme C			Scheme D		
Variant	***ST***	**Isolate (n)**	**%**	***ST***	**Isolate (n)**	**%**	***ST***	**Isolate (n)**	**%**	***ST***	**Isolate (n)**	**%**
L3 n= 3	*44*	2	100	*1*	2	100	*51*	1	100			
**Total**	***1***	**2**		***1***	**2**		***1***	**1**		***1***	**0**	

MLST	Scheme A			Scheme B			Scheme C			Scheme D		
Variant	***ST***	**Isolate (n)**	**%**	***ST***	**Isolate (n)**	**%**	***ST***	**Isolate (n)**	**%**	***ST***	**Isolate (n)**	**%**
LGV n= 2							*58*	2	100			
**Total**	***0***	**0**		***0***	**0**		***1***	**2**		***0***	**0**	

Supplementary Figure S4.

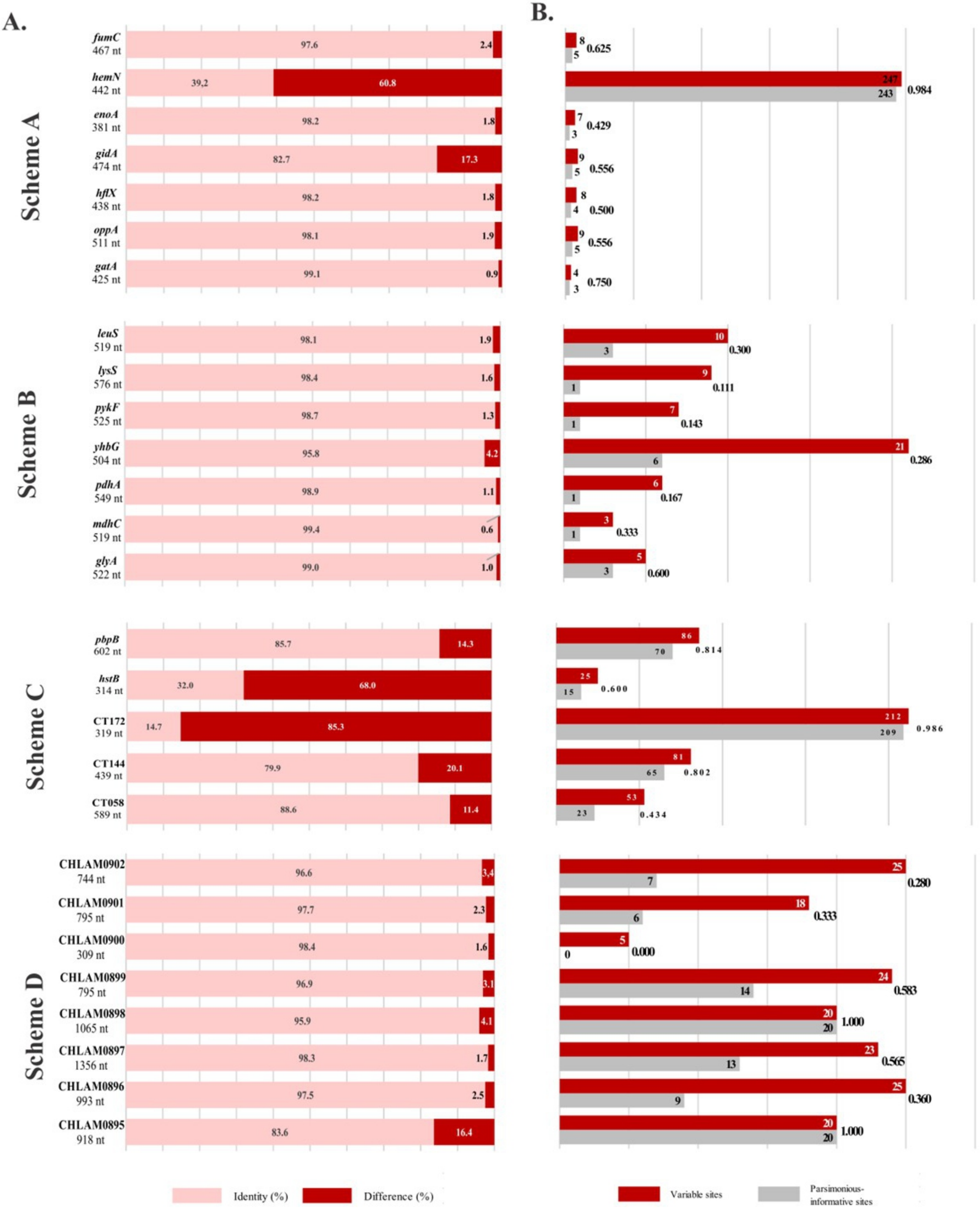

Supplementary Table S4.

A

Gene	Max length	Number of sequences used	Total number of sites (excluding sites with gaps / missing data)	Number of polymorphic (segregating) sites (S)	Total number of mutations (Eta)	Number of haplotypes (h)	Haplotype diversity (Hd)	Nucleotide diversity (π)	Theta (per site) from Eta	Theta (per site) from S (ThetaW)	Average number of nucleotide differences (k)
gatA	425	75	425	4	4	5	0.668 ± 0.041	0.00195	0.00193	0.00193 ± 0.00105	0.830
oppA	471	75	471	9	9	10	0.624± 0.053	0.00256	0.00391	0.00391 ± 0.00160	1.208
hflX	435	75	435	8	8	10	0.708± 0.037	0.00307	0.00376	0.00376 ± 0.00160	1.334
gidA	474	75	400	8	8	10	0.771± 0.027	0.00294	0.00409	0.00409 ± 0.00174	1.177
enoA	381	75	381	7	7	8	0.639± 0.039	0.00207	0.00376	0.00376± 0.00167	0.790
hemN	432	75	420	247	248	7	0.328± 0.066	0.03129	0.12080	0.12031± 0.03131	13.144
fumC	466	75	464	8	8	6	0.348± 0.067	0.00178	0.00353	0.00353 ± 0.00150	0.827
Concatenated	3084	75	2996	291	292	73	0.999± 0.002	0.00645	0.01994	0.00987± 0.00515	19.310

B

Gene	Max length	Number of sequences used	Total number of sites (excluding sites with gaps / missing data)	Number of polymorphic (segregating) sites (S)	Total number of mutations (Eta)	Number of haplotypes (h)	Haplotype diversity (Hd)	Nucleotide diversity (π)	Theta (per site) from Eta	Theta (per site) from S (ThetaW)	Average number of nucleotide differences (k)
glyA	522	7	522	5	5	7	1.000 ± 0.076	0.00401	0.00391	0.00391 ± 0.00234	2.095
mdhC	519	4	519	3	3	4	1.000 ± 0.177	0.00321	0.00315	0.00315 ± 0.00229	1.667
pdhA	549	7	549	6	6	7	1.000 ± 0.076	0.00347	0.00446	0.00446 ± 0.0257	1.905
yhbG	504	8	504	21	22	8	1.000 ± 0.063	0.01403	0.01684	0.01607 ± 0.00758	7.071
pykF	525	7	525	7	7	7	1.000 ± 0.076	0.00417	0.00544	0.00544 ± 0.00305	2.191
lysS	576	8	576	9	9	8	1.000 ± 0.063	0.00440	0.00603	0.00603 ± 0.00316	2.536
leuS	519	11	519	10	11	11	1.000 ± 0.039	0.00522	0.00724	0.00658 ± 0.00321	2.709
Concatenado	3729	44	3714	61	63	44	1.000 ± 0.005	0.00255	0.00390	0.00378 ± 0.00116	9.482

C

Gene	Max length	Number of sequences used	Total number of sites (excluding sites with gaps / missing data)	Number of polymorphic (segregating) sites (S)	Total number of mutations (Eta)	Number of haplotypes (h)	Haplotype diversity (Hd)	Nucleotide diversity (π)	Theta (per site) from Eta	Theta (per site) from S (ThetaW)	Average number of nucleotide differences (k)
CT058	605	53	589	53	55	44	0.990 ± 0.007	0.01086	0.02058	0.01983 ± 0.00597	6.399
CT144	448	30	439	81	83	30	1.000 ± 0.009	0.05683	0.04772	0.04657 ± 0.01504	24.949
CT172	708	41	319	212	246	13	0.801 ± 0.044	0.18962	0.18024	0.15533 ± 0.04546	60.489
hstB	909	92	314	25	26	19	0.829 ± 0.023	0.00997	0.01626	0.01563 ± 0.00487	3.132
pbpB	602	36	602	86	88	36	1.000 ± 0.007	0.02752	0.03525	0.03445 ± 0.01071	16.570
Concatenated	3138	183	2957	1737	2847	176	0.9995 ± 0.0007	0.22224	0.16646	0.10156 ± 0.02208	657.169

D

Gene	Max length	Number of sequences used	Total number of sites (excluding sites with gaps / missing data)	Number of polymorphic (segregating) sites (S)	Total number of mutations (Eta)	Number of haplotypes (h)	Haplotype diversity (Hd)	Nucleotide diversity (π)	Theta (per site) from Eta	Theta (per site) from S (ThetaW)	Average number of nucleotide differences (k)
CHLAM0895	918	16	381	16	16	10	0.933 ± 0.040	0.00454	0.00616	0.00616 ± 0.00261	3.558
CHLAM0896	993	18	993	25	25	18	1.000 ± 0.019	0.00565	0.00732	0.00732 ± 0.00287	5.608
CHLAM0897	1356	17	1356	23	23	17	1.000 ± 0.020	0.00436	0.00502	0.00502 ± 0.00201	5.912
CHLAM0898	1065	16	1041	21	22	15	0.992 ± 0.025	0.00572	0.00637	0.00608 ± 0.00249	5.958
CHLAM0899	795	13	795	24	26	13	1.000 ± 0.030	0.00950	0.01054	0.00973 ± 0.00408	7.551
CHLAM0900	309	6	309	5	5	6	1.000 ± 0.096	0.00539	0.00709	0.00709 ± 0.00434	1.667
CHLAM0901	795	16	795	18	19	16	1.000 ± 0.022	0.00498	0.00720	0.00682 ± 0.00285	3.958
CHLAM0902	744	22	744	25	26	22	1.000 ± 0.014	0.00539	0.00959	0.00922 ± 0.00349	4.013
Concatenated	6975	47	6963	147	152	46	0.9999 ± 0.005	0.00455	0.00494	0.00478 ± 0.00138	31.654

Supplementary Table S5.

Scheme A (Chlamydiales-*Ct*)

No. isolates = 75 | No. STs = 75 | No. re-samplings for bootstrapping = 1000

No. loci per isolate = 7 | No. identical loci for group def = 6 | No. groups = 3

Group 1: No. Isolates = 36 | No. STs = 36 | Predicted Founder = 13

ST	FREQ	SLV	DLV	TLV	SAT	Average Distance	ST Bootstrap Group	Subgrp
13	1	11	20	4	0	1.8	81%	97%
6	1	9	13	10	3	2.2	32%	92%
132	1	8	9	11	7	2.48	19%	83%
9	1	7	18	10	0	2.08	11%	67%
136	1	7	14	14	0	2.2	6%	61%
3	1	6	12	13	4	2.42	3%	50%
95	1	5	13	13	4	2.45	0%	13%
141	1	5	11	12	7	2.6	1%	20%
10	1	3	18	14	0	2.31	0%	0%
99	1	3	15	13	4	2.51	0%	0%
106	1	3	14	18	0	2.42	0%	0%
113	1	3	11	14	7	2.71	0%	0%
5	1	3	10	18	4	2.65	0%	0%
112	1	3	10	18	4	2.65	0%	0%
101	1	3	10	14	8	2.85	0%	2%
144	1	3	7	18	7	2.82	0%	0%
143	1	3	7	18	7	2.82	0%	0%
39	1	2	19	11	3	2.42	0%	0%
137	1	2	12	14	7	2.74	0%	0%
140	1	2	11	12	10	2.88	0%	0%
96	1	2	10	19	4	2.71	0%	0%
42	1	2	10	16	7	2.8	0%	0%
105	1	2	10	16	7	2.8	0%	0%
100	1	2	9	12	12	3.05	0%	0%
117	1	2	8	14	11	2.97	0%	0%
45	1	2	8	14	11	2.97	0%	0%
15	1	2	7	13	13	3.08	0%	0%
14	1	2	7	13	13	3.08	0%	0%
145	1	2	7	8	18	3.34	0%	0%
102	1	2	6	19	8	2.94	0%	0%
147	1	2	6	9	18	3.37	0%	0%
131	1	2	6	9	18	3.37	0%	0%
92	1	2	5	19	9	3.08	0%	0%
119	1	1	8	15	11	3.08	0%	0%
1	1	1	5	14	15	3.31	0%	0%
108	1	1	4	17	13	3.2	0%	0%

Group 2: No. Isolates = 26 | No. STs = 26 | Predicted Founder = 4								
						Average	ST Bootstrap	
ST	FREQ	SLV	DLV	TLV	SAT	Distance	Group	Subgrp
4	1	11	12	2	0	1.64	92%	98%
133	1	8	13	4	0	1.84	31%	89%
8	1	7	14	4	0	1.88	19%	67%
12	1	5	13	7	0	2.08	5%	26%
135	1	5	9	10	1	2.28	4%	26%
146	1	4	10	10	1	2.32	0%	4%
110	1	4	10	9	2	2.36	1%	9%
38	1	4	9	10	2	2.4	1%	12%
134	1	4	7	10	4	2.56	0%	8%
121	1	3	17	5	0	2.08	0%	0%
7	1	3	10	11	1	2.4	0%	0%
91	1	3	8	10	4	2.6	0%	2%
109	1	2	15	8	0	2.24	0%	0%
120	1	2	12	9	2	2.44	0%	0%
138	1	2	11	11	1	2.44	0%	0%
93	1	2	11	10	2	2.48	0%	0%
97	1	2	9	11	3	2.6	0%	0%
139	1	2	9	10	4	2.64	0%	0%
94	1	2	6	11	6	2.84	0%	0%
142	1	2	4	11	8	3.04	0%	0%
123	1	2	2	12	9	3.16	0%	0%
111	1	1	12	12	0	2.44	0%	0%
114	1	1	10	12	2	2.6	0%	0%
115	1	1	6	15	3	2.8	0%	0%
104	1	1	6	12	6	2.92	0%	0%
103	1	1	3	10	11	3.32	0%	0%

Group 3: No. Isolates = 7 | No. STs = 7 | Predicted Founder = 44								
						Average	ST Bootstrap	
ST	FREQ	SLV	DLV	TLV	SAT	Distance	Group	Subgrp
44	1	6	0	0	0	1.0	99%	93%
128	1	2	4	0	0	1.66	0%	0%
116	1	2	4	0	0	1.66	0%	0%
127	1	2	4	0	0	1.66	0%	0%
122	1	2	4	0	0	1.66	0%	0%
2	1	2	4	0	0	1.66	0%	0%
11	1	2	4	0	0	1.66	0%	0%

Singletons: size 6
126
125
124
118
107
98

Scheme B (C. trachomatis)

No. isolates = 44 | No. STs = 44 | No. re-samplings for bootstrapping = 1000

No. loci per isolate = 7 | No. identical loci for group def = 6 | No. groups = 3

Group 1: No. Isolates = 19 | No. STs = 19 | Predicted Founder = 19

ST	FREQ	SLV	DLV	TLV	SAT	Average Distance	ST Bootstrap Group	Subgrp
19	1	9	9	0	0	1.5	88%	98%
23	1	7	9	2	0	1.72	42%	87%
21	1	5	9	4	0	1.94	13%	42%
27	1	4	9	5	0	2.05	4%	18%
29	1	4	6	7	1	2.27	3%	8%
20	1	3	11	4	0	2.05	1%	0%
24	1	3	9	6	0	2.16	0%	5%
8	1	3	8	7	0	2.22	1%	0%
5	1	3	6	8	1	2.38	0%	0%
31	1	3	6	8	1	2.38	0%	0%
16	1	3	5	10	0	2.38	1%	4%
17	1	2	14	2	0	2.0	0%	0%
30	1	2	11	5	0	2.16	0%	0%
7	1	2	9	7	0	2.27	0%	0%
9	1	2	7	8	1	2.44	0%	0%
15	1	2	5	11	0	2.5	0%	0%
3	1	1	8	9	0	2.44	0%	0%
14	1	1	6	8	3	2.72	0%	0%
28	1	1	5	11	1	2.66	0%	0%

Group 2: No. Isolates = 7 | No. STs = 7 | Predicted Founder = 11

ST	FREQ	SLV	DLV	TLV	SAT	Average Distance	ST Bootstrap Group	Subgrp
11	1	3	3	0	0	1.5	49%	19%
43	1	3	2	1	0	1.66	36%	20%
13	1	2	3	1	0	1.83	10%	0%
44	1	2	2	2	0	2.0	9%	0%
12	1	2	2	2	0	2.0	8%	0%
42	1	1	2	2	1	2.5	0%	0%
10	1	1	2	2	1	2.5	0%	0%

Group 3: No. Isolates = 7 | No. STs = 7 | Predicted Founder = 34								
						Average	ST Bootstrap	
ST	FREQ	SLV	DLV	TLV	SAT	Distance	Group	Subgrp
34	1	4	2	0	0	1.33	71%	56%
39	1	3	3	0	0	1.5	32%	16%
36	1	2	3	1	0	1.83	4%	0%
40	1	2	2	2	0	2.0	9%	0%
38	1	1	3	2	0	2.16	0%	0%
35	1	1	3	2	0	2.16	0%	0%
32	1	1	2	3	0	2.33	0%	0%

Singletons: size 11
26
25
22
18
6
4
2
1
41
37
33

Scheme C (C. trachomatis Uppsala)

No. isolates = 520 | No. STs = 520 | No. re-samplings for bootstrapping = 1000

No. loci per isolate = 5 | No. identical loci for group def = 4 | No. groups = 15

Group 1: No. Isolates = 241 | No. STs = 241 | Predicted Founder = 56

ST	FREQ	SLV	DLV	TLV	SAT	Average Distance	ST Bootstrap Group	Subgrp
56	1	37	52	79	72	2.80	88%	100%
236	1	32	81	72	55	2.64	36%	99%
59	1	30	51	71	88	2.94	11%	98%
3	1	28	23	51	138	3.46	13%	100%
12	1	27	33	78	102	3.23	8%	100%
153	1	25	56	72	87	2.96	0%	36%
64	1	25	55	73	87	2.96	0%	38%
504	1	24	57	71	88	2.97	0%	9%
513	1	23	59	73	85	2.95	0%	0%
171	1	23	58	74	85	2.96	0%	0%
73	1	23	57	73	87	2.97	0%	0%
214	1	23	57	72	88	2.97	0%	0%
154	1	23	57	72	88	2.97	0%	0%
497	1	23	57	72	88	2.97	0%	0%
496	1	23	57	72	88	2.97	0%	0%
70	1	23	57	72	88	2.97	0%	0%
334	1	23	57	72	88	2.97	0%	0%
327	1	22	60	71	87	2.97	0%	0%
310	1	22	58	73	87	2.97	0%	0%
515	1	22	58	73	87	2.97	0%	0%
323	1	22	58	72	88	2.98	0%	0%
184	1	22	58	72	88	2.98	0%	0%
114	1	22	58	72	88	2.98	0%	0%
377	1	22	58	72	88	2.98	0%	0%
331	1	22	58	72	88	2.98	0%	0%
148	1	19	99	70	52	2.66	0%	98%
237	1	18	78	112	32	2.66	0%	90%
454	1	17	35	65	123	3.30	0%	53%
319	1	17	24	45	154	3.62	0%	64%
510	1	16	27	43	154	3.61	0%	31%
109	1	16	8	34	182	3.71	0%	100%
77	1	15	70	96	59	2.85	0%	96%
172	1	15	60	77	88	3.04	0%	93%
326	1	15	27	44	154	3.62	0%	4%
450	1	14	74	97	55	2.82	0%	64%
110	1	14	39	116	71	3.04	0%	95%
87	1	14	29	45	152	3.62	0%	1%
305	1	14	28	44	154	3.63	0%	1%
426	1	14	27	47	152	3.62	0%	0%
330	1	14	27	45	154	3.63	0%	1%
329	1	14	27	44	155	3.64	0%	1%
328	1	14	27	44	155	3.64	0%	1%
325	1	14	27	44	155	3.64	0%	1%
155	1	14	27	44	155	3.64	0%	1%
434	1	14	27	44	155	3.64	0%	1%
396	1	14	27	44	155	3.64	0%	1%
147	1	13	73	104	50	2.82	0%	25%
157	1	12	60	74	94	3.21	0%	26%
11	1	11	77	96	56	2.84	0%	30%
419	1	11	58	98	73	3.00	0%	15%
174	1	11	44	80	105	3.23	0%	18%
91	1	11	43	105	81	3.10	0%	80%
79	1	11	37	76	116	3.42	0%	17%
179	1	10	51	136	43	2.88	0%	39%
62	1	10	47	79	104	3.32	0%	64%
69	1	9	44	110	77	3.08	0%	25%
461	1	9	33	81	117	3.45	0%	3%
158	1	8	62	120	50	2.88	0%	4%
428	1	8	58	116	58	2.96	0%	0%
452	1	8	55	102	75	3.05	0%	0%
262	1	8	46	91	95	3.18	0%	48%

342	1	8	43	95	94	3.19	0%	14%
516	1	8	40	116	76	3.10	0%	24%
231	1	8	36	112	84	3.17	0%	31%
193	1	8	35	88	109	3.40	0%	0%
460	1	8	35	80	117	3.45	0%	0%
198	1	8	34	84	114	3.45	0%	0%
235	1	8	34	81	117	3.46	0%	0%
90	1	8	29	44	159	3.74	0%	32%
216	1	8	28	82	122	3.56	0%	29%
146	1	7	77	97	59	2.89	0%	0%
462	1	7	77	97	59	2.89	0%	0%
451	1	7	58	100	75	3.04	0%	0%
432	1	7	57	117	59	2.98	0%	0%
226	1	7	56	102	75	3.05	0%	0%
388	1	7	56	102	75	3.05	0%	0%
1	1	7	47	76	110	3.26	0%	23%
445	1	7	41	52	140	3.55	0%	8%
421	1	7	38	83	112	3.44	0%	24%
530	1	7	29	83	121	3.56	0%	16%
512	1	7	27	124	82	3.19	0%	8%
475	1	7	17	103	113	3.4	0%	47%
357	1	6	73	93	68	2.96	0%	0%
505	1	6	57	118	59	2.99	0%	0%
392	1	6	56	119	59	2.99	0%	0%
89	1	6	54	82	98	3.30	0%	0%
466	1	6	52	80	102	3.33	0%	0%
457	1	6	50	114	70	3.06	0%	1%
312	1	6	48	99	87	3.16	0%	0%
391	1	6	47	99	88	3.17	0%	4%
447	1	6	40	95	99	3.25	0%	5%
458	1	6	39	128	67	3.08	0%	0%
535	1	6	37	113	84	3.17	0%	5%
188	1	6	32	91	111	3.32	0%	41%
297	1	6	32	81	121	3.55	0%	5%
337	1	6	30	82	122	3.57	0%	5%
336	1	6	30	82	122	3.57	0%	6%
332	1	6	30	82	122	3.57	0%	5%
7	1	6	30	49	155	3.69	0%	14%
414	1	6	29	122	83	3.2	0%	4%
80	1	6	29	45	160	3.72	0%	15%
416	1	6	27	124	83	3.20	0%	4%
415	1	6	27	124	83	3.20	0%	4%
418	1	6	24	127	83	3.22	0%	5%
420	1	6	24	100	110	3.36	0%	33%
242	1	6	24	66	144	3.54	0%	18%
243	1	6	17	35	182	3.76	0%	18%
537	1	5	69	97	69	2.99	0%	0%
225	1	5	56	75	104	3.22	0%	2%
238	1	5	49	81	105	3.36	0%	0%
400	1	5	49	81	105	3.36	0%	0%
364	1	5	48	74	113	3.29	0%	4%
195	1	5	47	102	86	3.17	0%	0%
233	1	5	38	51	146	3.67	0%	0%
469	1	5	36	68	131	3.61	0%	5%
186	1	5	32	105	98	3.28	0%	1%
240	1	5	29	44	162	3.79	0%	1%
509	1	5	27	50	158	3.70	0%	0%
234	1	5	25	118	92	3.27	0%	0%
474	1	5	19	113	103	3.35	0%	4%
14	1	5	18	34	183	3.77	0%	3%
244	1	5	18	33	184	3.77	0%	4%
382	1	5	18	33	184	3.77	0%	4%
386	1	5	17	83	135	3.60	0%	6%
473	1	4	56	89	91	3.16	0%	0%
464	1	4	51	86	99	3.33	0%	0%
163	1	4	49	74	113	3.3	0%	0%
366	1	4	49	74	113	3.3	0%	0%
529	1	4	46	97	93	3.21	0%	0%
453	1	4	44	75	117	3.47	0%	0%

189	1	4	43	78	115	3.46	0%	0%
448	1	4	41	52	143	3.60	0%	0%
197	1	4	40	99	97	3.25	0%	0%
397	1	4	40	97	99	3.27	0%	0%
378	1	4	40	53	143	3.61	0%	0%
85	1	4	37	123	76	3.15	0%	0%
547	1	4	36	57	143	3.64	0%	0%
6	1	4	34	75	127	3.42	0%	0%
422	1	4	33	53	150	3.68	0%	0%
465	1	4	32	95	109	3.35	0%	0%
45	1	4	32	76	128	3.44	0%	0%
423	1	4	32	55	149	3.68	0%	0%
248	1	4	32	44	160	3.72	0%	0%
467	1	4	31	86	119	3.52	0%	0%
251	1	4	31	45	160	3.72	0%	0%
13	1	4	30	44	162	3.80	0%	0%
538	1	4	30	22	184	4.02	0%	2%
222	1	4	29	49	158	3.73	0%	11%
536	1	4	28	50	158	3.70	0%	0%
424	1	4	28	23	185	4.15	0%	6%
160	1	4	26	51	159	3.71	0%	0%
224	1	4	25	119	92	3.26	0%	0%
399	1	4	18	58	160	3.83	0%	0%
318	1	4	14	14	208	4.04	0%	10%
324	1	4	14	12	210	4.05	0%	9%
9	1	4	13	67	156	3.79	0%	1%
455	1	4	13	61	162	3.81	0%	2%
311	1	4	13	14	209	4.05	0%	10%
181	1	4	13	13	210	4.05	0%	9%
252	1	3	52	65	120	3.51	0%	0%
253	1	3	49	98	90	3.19	0%	0%
16	1	3	36	61	140	3.50	0%	0%
508	1	3	35	85	117	3.4	0%	0%
239	1	3	33	94	110	3.36	0%	0%
5	1	3	33	76	128	3.44	0%	0%
385	1	3	33	75	129	3.62	0%	0%
514	1	3	32	77	128	3.62	0%	0%
228	1	3	31	81	125	3.44	0%	0%
375	1	3	31	49	157	3.72	0%	0%
213	1	3	30	104	103	3.30	0%	0%
365	1	3	30	103	104	3.33	0%	0%
356	1	3	30	48	159	3.74	0%	0%
498	1	3	29	67	141	3.54	0%	0%
67	1	3	29	67	141	3.54	0%	3%
86	1	3	29	24	184	4.15	0%	0%
463	1	3	28	87	122	3.55	0%	0%
446	1	3	27	69	141	3.60	0%	0%
194	1	3	20	35	182	3.77	0%	0%
180	1	3	20	35	182	3.77	0%	0%
207	1	3	20	35	182	3.77	0%	0%

84	1	3	18	118	101	3.34	0%	0%
230	1	3	17	121	99	3.34	0%	0%
76	1	3	17	119	101	3.35	0%	0%
441	1	3	15	58	164	3.87	0%	0%
166	1	3	14	100	123	3.5	0%	0%
250	1	3	13	62	162	3.82	0%	0%
8	1	3	12	32	193	4.20	0%	0%
503	1	2	53	97	88	3.17	0%	0%
427	1	2	47	101	90	3.21	0%	0%
370	1	2	46	69	123	3.37	0%	0%
255	1	2	40	67	131	3.44	0%	0%
61	1	2	40	67	131	3.44	0%	0%
394	1	2	36	84	118	3.40	0%	0%
152	1	2	35	72	131	3.46	0%	0%
187	1	2	29	116	93	3.27	0%	0%
507	1	2	25	84	129	3.60	0%	0%
522	1	2	24	61	153	3.60	0%	0%
241	1	2	23	100	115	3.42	0%	0%
338	1	2	19	106	113	3.43	0%	0%
387	1	2	19	62	157	3.74	0%	0%
519	1	2	18	113	107	3.38	0%	0%
333	1	2	16	106	116	3.44	0%	0%
321	1	2	15	99	124	3.51	0%	0%
528	1	2	15	75	148	3.65	0%	0%
246	1	2	15	14	209	4.38	0%	0%
245	1	2	15	11	212	4.41	0%	0%
533	1	2	14	74	150	3.66	0%	0%
398	1	2	14	67	157	3.74	0%	0%
247	1	2	14	34	190	3.84	0%	0%
129	1	2	13	100	125	3.69	0%	0%
521	1	2	12	72	154	3.65	0%	0%
511	1	2	11	71	156	3.76	0%	0%
18	1	2	10	57	171	3.87	0%	0%
19	1	2	7	54	177	3.93	0%	0%
520	1	2	6	55	177	4.02	0%	0%
313	1	1	37	72	130	3.45	0%	0%
55	1	1	35	73	131	3.48	0%	0%
374	1	1	34	74	131	3.47	0%	0%
320	1	1	31	76	132	3.66	0%	0%
215	1	1	31	67	141	3.55	0%	0%
66	1	1	31	67	141	3.55	0%	0%
506	1	1	24	46	169	3.83	0%	0%
491	1	1	17	78	144	3.78	0%	0%
390	1	1	17	64	158	3.8	0%	0%
431	1	1	17	28	194	4.33	0%	0%
531	1	1	16	79	144	3.79	0%	0%
459	1	1	14	69	156	3.75	0%	0%
468	1	1	14	57	168	3.92	0%	0%
478	1	1	13	38	188	4.04	0%	0%
456	1	1	12	61	166	3.80	0%	0%
379	1	1	11	88	140	3.62	0%	0%
229	1	1	10	84	145	3.63	0%	0%
44	1	1	10	84	145	3.63	0%	0%
298	1	1	10	80	149	3.83	0%	0%
425	1	1	9	42	188	4.14	0%	0%
223	1	1	8	64	167	3.87	0%	0%
182	1	1	7	25	207	4.42	0%	0%
254	1	1	6	46	187	4.10	0%	0%
476	1	1	6	28	205	4.06	0%	0%
10	1	1	3	30	206	4.18	0%	0%
532	1	1	3	15	221	4.35	0%	0%

Group 2: No. Isolates = 167 | No. STs = 167 | Predicted Founder = 108

ST	FREQ	SLV	DLV	TLV	SAT	Average Distance	ST Bootstrap Group	Subgrp
108	1	17	12	17	120	3.81	52%	97%
52	1	15	14	17	120	3.83	19%	91%
33	1	14	28	46	78	3.25	14%	78%
35	1	14	27	47	78	3.23	25%	97%
20	1	13	31	38	84	3.39	17%	91%
136	1	12	19	34	101	3.47	5%	83%
135	1	12	15	40	99	3.57	5%	87%
34	1	11	34	49	72	3.19	4%	75%
106	1	11	24	50	81	3.46	3%	80%
276	1	11	14	18	123	3.78	0%	66%
26	1	10	39	38	79	3.35	1%	41%
130	1	10	33	49	74	3.24	1%	66%
281	1	10	30	60	66	3.13	0%	71%
30	1	10	26	46	84	3.34	1%	80%
202	1	10	18	18	120	3.86	0%	6%
354	1	10	18	18	120	3.86	0%	6%
353	1	10	18	18	120	3.86	0%	5%
352	1	10	18	18	120	3.86	0%	6%
346	1	10	18	18	120	3.86	0%	5%
345	1	10	18	18	120	3.86	0%	5%
344	1	10	18	18	120	3.86	0%	6%
343	1	10	18	18	120	3.86	0%	6%
268	1	10	17	27	112	3.68	0%	38%
27	1	10	6	28	122	3.78	0%	98%
266	1	9	38	42	77	3.34	0%	28%
220	1	9	37	42	78	3.25	0%	35%
32	1	9	31	42	84	3.44	0%	54%
37	1	9	30	74	53	3.09	0%	43%
24	1	9	29	43	85	3.46	0%	33%
484	1	9	25	53	79	3.33	0%	66%
100	1	9	18	27	112	3.69	0%	5%
274	1	9	15	18	124	3.81	0%	5%
273	1	9	15	17	125	3.81	0%	5%
404	1	8	38	51	69	3.19	0%	10%
128	1	8	36	53	69	3.18	0%	9%
82	1	8	34	44	80	3.29	0%	15%
402	1	8	28	50	80	3.45	0%	25%
279	1	8	27	52	79	3.43	0%	32%
264	1	8	26	61	71	3.29	0%	40%
265	1	8	25	51	82	3.36	0%	15%
134	1	8	21	47	90	3.57	0%	22%
272	1	8	16	17	125	3.82	0%	0%
269	1	8	16	17	125	3.82	0%	0%
95	1	7	34	76	49	3.07	0%	10%
381	1	7	33	69	57	3.13	0%	15%
540	1	7	31	41	87	3.51	0%	7%
25	1	7	28	56	75	3.30	0%	7%
208	1	7	22	66	71	3.24	0%	21%
22	1	7	20	51	88	3.56	0%	10%
517	1	7	18	11	130	4.04	0%	40%
111	1	6	31	52	77	3.30	0%	2%
477	1	6	30	51	79	3.40	0%	2%
285	1	6	28	53	79	3.35	0%	59%
541	1	6	27	53	80	3.34	0%	1%
105	1	6	26	56	78	3.39	0%	6%
380	1	6	26	46	88	3.56	0%	3%
133	1	6	23	51	86	3.54	0%	4%
94	1	6	22	36	102	3.52	0%	22%
518	1	6	17	10	133	4.15	0%	9%
315	1	6	17	6	137	4.35	0%	15%
28	1	6	12	48	100	3.57	0%	37%
156	1	5	34	80	47	3.03	0%	0%
65	1	5	33	44	84	3.48	0%	0%
93	1	5	31	51	79	3.32	0%	0%
408	1	5	31	51	79	3.32	0%	0%

486	1	5	28	63	70	3.29	0%	0%
372	1	5	26	55	80	3.48	0%	2%
211	1	5	23	70	68	3.23	0%	1%
102	1	5	23	54	84	3.54	0%	2%
137	1	5	19	61	81	3.45	0%	38%
112	1	5	18	27	116	3.91	0%	9%
209	1	5	18	22	121	4.01	0%	4%
101	1	5	17	10	134	4.26	0%	6%
301	1	5	16	24	121	4.03	0%	7%
29	1	5	13	37	111	3.68	0%	12%
17	1	5	6	34	121	4.03	0%	24%
545	1	4	32	54	76	3.38	0%	0%
190	1	4	28	52	82	3.45	0%	1%
410	1	4	26	46	90	3.57	0%	0%
291	1	4	25	47	90	3.57	0%	0%
38	1	4	22	60	80	3.41	0%	0%
525	1	4	21	61	80	3.42	0%	0%
270	1	4	20	45	97	3.53	0%	1%
550	1	4	17	51	94	3.66	0%	0%
219	1	4	13	42	107	3.72	0%	2%
287	1	4	11	61	90	3.57	0%	9%
373	1	4	11	28	123	3.79	0%	1%
358	1	4	10	50	102	3.63	0%	2%
278	1	4	9	16	137	3.92	0%	1%
103	1	4	5	32	125	4.05	0%	54%
277	1	3	29	80	54	3.17	0%	0%
485	1	3	26	49	88	3.57	0%	0%
31	1	3	24	50	89	3.46	0%	0%
258	1	3	22	17	124	3.98	0%	0%
409	1	3	20	56	87	3.48	0%	0%
304	1	3	20	26	117	3.93	0%	0%
367	1	3	19	22	122	3.95	0%	0%
407	1	3	18	54	91	3.63	0%	0%
308	1	3	18	14	131	4.16	0%	0%
351	1	3	17	9	137	4.39	0%	0%
275	1	3	16	28	119	3.83	0%	0%
169	1	3	16	17	130	4.13	0%	0%
348	1	3	16	14	133	4.19	0%	0%
490	1	3	13	49	101	3.64	0%	0%
257	1	3	13	35	115	3.98	0%	0%
355	1	3	13	35	115	4.00	0%	0%
161	1	3	12	37	114	3.74	0%	5%
471	1	3	12	33	118	3.83	0%	9%
412	1	3	11	28	124	3.87	0%	0%
500	1	3	11	27	125	4.09	0%	0%
259	1	3	9	50	104	3.64	0%	0%
435	1	3	9	14	140	4.29	0%	0%
524	1	3	7	58	98	3.69	0%	0%
501	1	3	6	21	136	4.21	0%	3%
317	1	3	5	15	143	4.51	0%	2%
316	1	3	4	18	141	4.42	0%	2%
36	1	2	27	53	84	3.43	0%	0%
413	1	2	21	83	60	3.27	0%	0%
406	1	2	20	62	82	3.56	0%	0%
260	1	2	20	33	111	3.86	0%	0%
309	1	2	20	22	122	3.95	0%	0%
284	1	2	19	70	75	3.38	0%	0%
306	1	2	19	24	121	3.91	0%	0%
54	1	2	19	23	122	3.96	0%	0%
401	1	2	18	32	114	3.96	0%	0%
307	1	2	18	15	131	4.10	0%	0%
539	1	2	17	39	108	3.68	0%	0%
168	1	2	16	56	92	3.67	0%	0%
389	1	2	16	40	108	3.74	0%	0%
481	1	2	16	40	108	3.70	0%	0%
303	1	2	16	17	131	4.12	0%	0%
302	1	2	16	17	131	4.12	0%	0%
350	1	2	16	11	137	4.42	0%	0%
286	1	2	15	58	91	3.56	0%	0%

479	1	2	14	49	101	3.69	0%	0%
81	1	2	14	35	115	4.01	0%	0%
282	1	2	13	49	102	3.66	0%	0%
436	1	2	12	28	124	3.87	0%	0%
221	1	2	11	36	117	3.79	0%	0%
527	1	2	11	34	119	4.01	0%	0%
74	1	2	11	32	121	3.75	0%	0%
127	1	2	10	34	120	4.07	0%	0%
395	1	2	9	40	115	3.90	0%	0%
359	1	2	9	31	124	4.09	0%	0%
217	1	2	9	9	146	4.39	0%	0%
292	1	2	8	45	111	3.77	0%	0%
176	1	2	5	35	124	4.06	0%	0%
472	1	2	4	26	134	4.14	0%	0%
534	1	1	23	78	64	3.32	0%	0%
201	1	1	22	65	78	3.40	0%	0%
489	1	1	22	52	91	3.54	0%	0%
290	1	1	20	57	88	3.63	0%	0%
411	1	1	20	53	92	3.65	0%	0%
92	1	1	19	67	79	3.46	0%	0%
322	1	1	17	11	137	4.42	0%	0%
271	1	1	16	41	108	3.76	0%	0%
261	1	1	16	37	112	3.90	0%	0%
15	1	1	14	39	112	3.85	0%	0%
363	1	1	12	45	108	3.95	0%	0%
482	1	1	12	31	122	3.94	0%	0%
256	1	1	10	47	108	3.80	0%	0%
203	1	1	10	31	124	3.95	0%	0%
140	1	1	9	37	119	4.06	0%	0%
288	1	1	7	33	125	3.86	0%	0%
191	1	1	5	35	125	4.18	0%	0%
369	1	1	5	23	137	4.19	0%	0%
218	1	1	3	45	117	3.88	0%	0%

Group 3: No. Isolates = 21 | No. STs = 21 | Predicted Founder = 97

ST	FREQ	SLV	DLV	TLV	SAT	Average Distance	ST Bootstrap Group	Subgrp
97	1	8	6	5	1	1.95	85%	97%
165	1	5	11	4	0	1.95	30%	64%
104	1	4	10	4	2	2.2	12%	29%
41	1	4	9	6	1	2.2	15%	31%
442	1	4	6	9	1	2.35	5%	10%
296	1	3	9	6	2	2.35	0%	0%
494	1	3	8	8	1	2.35	0%	0%
294	1	3	8	8	1	2.35	0%	5%
164	1	3	7	10	0	2.35	1%	7%
443	1	3	7	9	1	2.4	0%	0%
42	1	3	6	9	2	2.5	3%	12%
492	1	2	8	10	0	2.4	0%	0%
548	1	2	7	8	3	2.6	0%	0%
493	1	2	3	13	2	2.75	0%	0%
43	1	2	3	9	6	3.0	0%	0%
295	1	2	1	13	4	2.95	0%	0%
444	1	1	8	5	6	2.85	0%	0%
96	1	1	7	7	5	2.8	0%	0%
200	1	1	6	10	3	2.75	0%	0%
542	1	1	4	9	6	3.1	0%	0%
167	1	1	2	6	11	3.45	0%	0%

Group 4: No. Isolates = 6 | No. STs = 6 | Predicted Founder = 205

ST	FREQ	SLV	DLV	TLV	SAT	Average Distance	ST Bootstrap Group	Subgrp
205	1	5	0	0	0	1.0	98%	82%
175	1	2	3	0	0	1.6	0%	0%
483	1	2	3	0	0	1.6	0%	0%
173	1	1	4	0	0	1.8	0%	0%
204	1	1	4	0	0	1.8	0%	0%
293	1	1	4	0	0	1.8	0%	0%

Group 5: No. Isolates = 5 | No. STs = 5 | Predicted Founder = Multiple Candidates

ST	FREQ	SLV	DLV	TLV	SAT	Average Distance	ST Bootstrap Group	Subgrp
115	1	3	1	0	0	1.25	43%	16%
47	1	3	1	0	0	1.25	47%	19%
118	1	2	2	0	0	1.5	3%	0%
116	1	1	3	0	0	1.75	0%	0%
117	1	1	3	0	0	1.75	0%	0%

Group 6: No. Isolates = 4 | No. STs = 4 | Predicted Founder = 98

ST	FREQ	SLV	DLV	TLV	SAT	Average Distance	ST Bootstrap Group	Subgrp
98	1	3	0	0	0	1.0	75%	12%
314	1	1	2	0	0	1.66	0%	0%
99	1	1	2	0	0	1.66	0%	0%
393	1	1	2	0	0	1.66	0%	0%

Group 7: No. Isolates = 3 | No. STs = 3 | Predicted Founder = 125

ST	FREQ	SLV	DLV	TLV	SAT	Average Distance	ST Bootstrap Group	Subgrp
125	1	2	0	0	0	1.0	32%	0%
126	1	1	1	0	0	1.5	0%	0%
120	1	1	1	0	0	1.5	0%	0%

Group 8: No. Isolates = 3 | No. STs = 3 | Predicted Founder = Multiple Candidates

ST	FREQ	SLV	DLV	TLV	SAT	Average Distance	ST Bootstrap Group	Subgrp
470	1	2	0	0	0	1.0	7%	0%
430	1	2	0	0	0	1.0	8%	0%
429	1	2	0	0	0	1.0	14%	0%

Group 9: No. Isolates = 3 | No. STs = 3 | Predicted Founder = 439

ST	FREQ	SLV	DLV	TLV	SAT	Average Distance	ST Bootstrap Group	Subgrp
439	1	2	0	0	0	1.0	31%	0%
440	1	1	1	0	0	1.5	0%	0%
438	1	1	1	0	0	1.5	0%	0%

Group 10: No. Isolates = 2 | No. STs = 2 | Predicted Founder = None

ST	FREQ	SLV	DLV	TLV	SAT	Distance
144	1	1	0	0	0	1.0
141	1	1	0	0	0	1.0

Group 11: No. Isolates = 2 | No. STs = 2 | Predicted Founder = None

ST	FREQ	SLV	DLV	TLV	SAT	Distance
143	1	1	0	0	0	1.0
58	1	1	0	0	0	1.0

Group 12: No. Isolates = 2 | No. STs = 2 | Predicted Founder = None

ST	FREQ	SLV	DLV	TLV	SAT	Distance
138	1	1	0	0	0	1.0
40	1	1	0	0	0	1.0

Group 13: No. Isolates = 2 | No. STs = 2 | Predicted Founder = None

ST	FREQ	SLV	DLV	TLV	SAT	Distance
488	1	1	0	0	0	1.0
487	1	1	0	0	0	1.0

Group 14: No. Isolates = 2 | No. STs = 2 | Predicted Founder = None

ST	FREQ	SLV	DLV	TLV	SAT	Distance
124	1	1	0	0	0	1.0
123	1	1	0	0	0	1.0

Group 15: No. Isolates = 2 | No. STs = 2 | Predicted Founder = None

ST	FREQ	SLV	DLV	TLV	SAT	Distance
122	1	1	0	0	0	1.0
119	1	1	0	0	0	1.0

Singletons: size 55

289	549	433	361
283	546	68	360
280	544	384	349
300	543	383	347
267	142	57	4
263	499	51	341
249	495	417	340
232	526	376	339
227	523	371	335
183	480	49	299
212	502	48	
178	449	46	
177	78	405	
206	75	368	
159	437	362	

Scheme D (Plasmid loci)

No. isolates = 47 | No. STs = 47 | No. re-samplings for bootstrapping = 1000
No. loci per isolate = 8 | No. identical loci for group def = 7 | No. groups = 6

Group 1: No. Isolates = 12 | No. STs = 12 | Predicted Founder = 2

ST	FREQ	SLV	DLV	TLV	SAT	Average Distance	ST Bootstrap Group	Subgrp
2	1	7	2	2	0	1.54	94%	96%
126	1	3	8	0	0	1.72	13%	25%
8	1	3	4	4	0	2.09	21%	10%
13	1	2	6	3	0	2.09	0%	0%
94	1	2	6	3	0	2.09	0%	0%
86	1	2	5	3	1	2.27	0%	0%
12	1	2	5	3	1	2.27	0%	0%
112	1	1	7	1	2	2.36	0%	0%
40	1	1	6	2	2	2.45	0%	0%
122	1	1	3	7	0	2.54	0%	0%
53	1	1	2	6	2	2.81	0%	0%
51	1	1	2	4	4	3.0	0%	0%

Group 2: No. Isolates = 9 | No. STs = 9 | Predicted Founder = 9

ST	FREQ	SLV	DLV	TLV	SAT	Average Distance	ST Bootstrap Group	Subgrp
9	1	8	0	0	0	1.0	99%	99%
135	1	3	5	0	0	1.62	4%	0%
102	1	3	5	0	0	1.62	4%	0%
73	1	3	5	0	0	1.62	8%	0%
6	1	2	6	0	0	1.75	0%	0%
41	1	2	6	0	0	1.75	0%	0%
138	1	1	7	0	0	1.87	0%	0%
136	1	1	7	0	0	1.87	0%	0%
113	1	1	7	0	0	1.87	0%	0%

Group 3: No. Isolates = 4 | No. STs = 4 | Predicted Founder = 4

ST	FREQ	SLV	DLV	TLV	SAT	Average Distance	ST Bootstrap Group	Subgrp
4	1	3	0	0	0	1.0	77%	14%
100	1	1	2	0	0	1.66	0%	0%
1	1	1	2	0	0	1.66	0%	0%
36	1	1	2	0	0	1.66	0%	0%

Group 4: No. Isolates = 4 | No. STs = 4 | Predicted Founder = 68

ST	FREQ	SLV	DLV	TLV	SAT	Average Distance	ST Bootstrap Group	Subgrp
68	1	3	0	0	0	1.0	72%	12%
76	1	1	2	0	0	1.66	0%	0%
69	1	1	2	0	0	1.66	0%	0%
143	1	1	2	0	0	1.66	0%	0%

Group 5: No. Isolates = 3 | No. STs = 3 | Predicted Founder = 21								
						Average	ST Bootstrap	
ST	FREQ	SLV	DLV	TLV	SAT	Distance	Group	Subgrp
21	1	2	0	0	0	1.0	32%	0%
3	1	1	1	0	0	1.5	0%	0%
31	1	1	1	0	0	1.5	0%	0%

Group 6: No. Isolates = 3 | No. STs = 3 | Predicted Founder = 117								
						Average	ST Bootstrap	
ST	FREQ	SLV	DLV	TLV	SAT	Distance	Group	Subgrp
117	1	2	0	0	0	1.0	28%	0%
80	1	1	1	0	0	1.5	0%	0%
140	1	1	1	0	0	1.5	0%	0%

Singletons: size 12
103
132
19
16
121
74
70
148
38
33
62
90

Supplementary Figure S5.

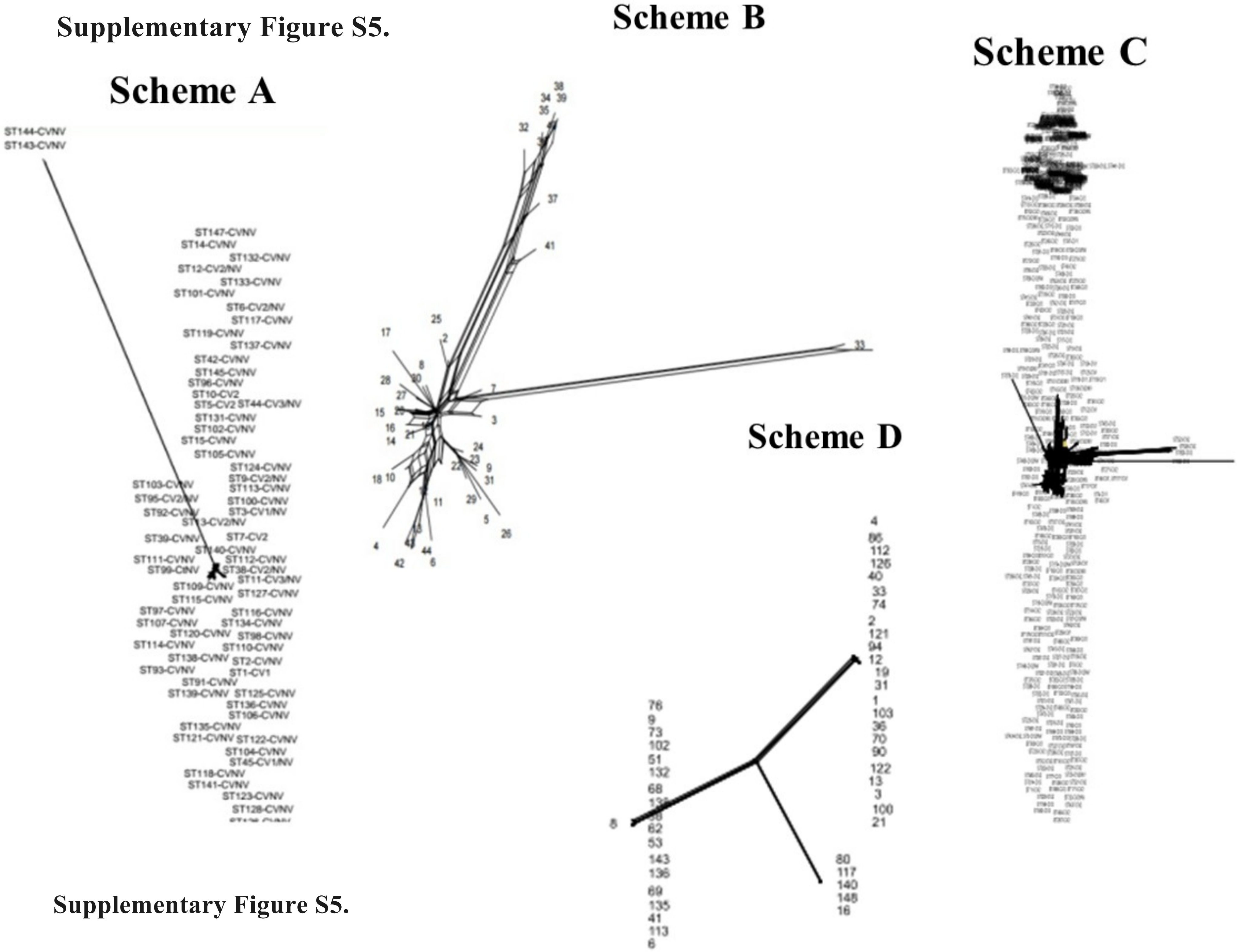

Supplementary Figure S5.

Supplementary Figure S6.

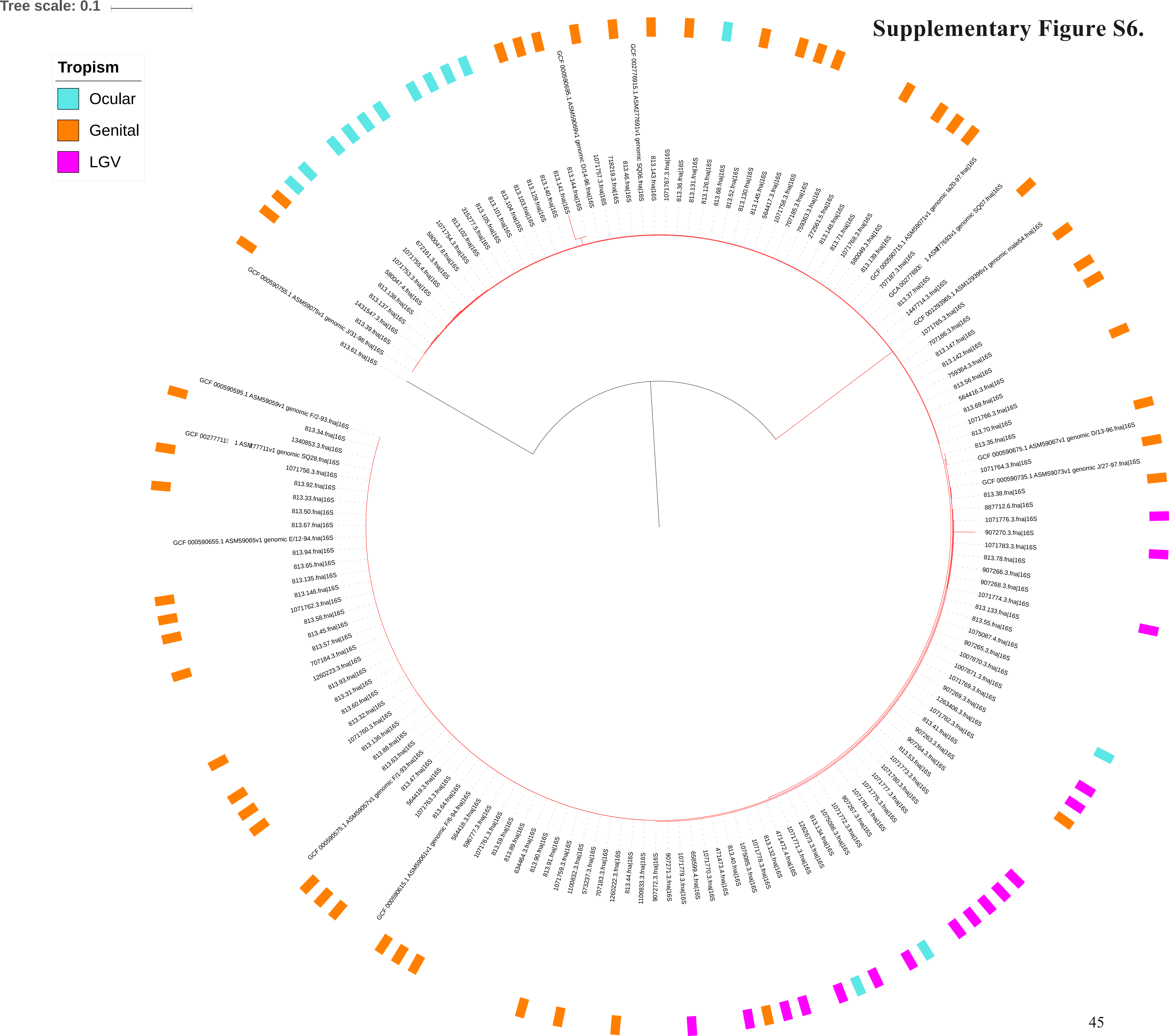

Supplementary Table S6.

Number of Loci	Maximum number of STs	Number of combinations analyzed	Molecular Marker (s)
1	21	19	1-hctB
2	39	171	1-gidA, -hctB
3	56	969	1-gidA, -hctB, -pbpB
4	64	3876	1-gidA, -leuS, -hctB, -pbpB
5	69	11628	1-gidA, -lysS, -CT058, -CT172, -hctB<br>2-gidA, -leuS, -CT058, -CT172, -hctB<br>3-gidA, -leuS, -CT058, -hctB, -pbpB<br>4-gidA, -leuS, -CT172, -hctB, -pbpB
6	72	27132	1-gidA, -mdhc, -lysS, -CT058, -CT172, -hctB<br>2-gidA, -mdhc, -lysS, -CT058, -hctB, -pbpB<br>3-gidA, -pykF, -lysS, -CT058, -CT172, -hctB<br>4-gidA, -lysS, -leuS, -CT058, -CT172, -hctB<br>5-gidA, -leuS, -CT058, -CT172, -hctB, -pbpB
7	74	50388	1-gidA, -mdhc, -lysS, -CT058, -CT172, -hctB, -pbpB<br>2-gidA, -pykF, -lysS, -CT058, -CT172, -hctB, -pbpB<br>3-gidA, -pykF, -leuS, -CT058, -CT172, -hctB, -pbpB<br>4-gidA, -lysS, -leuS, -CT058, -CT172, -hctB, -pbpB,
8	75	75582	1-hflX, -gidA, -mdhc, -lysS, -CT058, -CT172, -hctB, -pbpB<br>2-hflX, -gidA, -pykF, -lysS, -CT058, -CT172, -hctB, -pbpB<br>3-hflX, -gidA, -pykF, -leuS, -CT058, -CT172, -hctB, -pbpB,<br>4-hflX, -gidA, -lysS, -leuS, -CT058, -CT172, -hctB, -pbpB<br>5-gidA, -hemN, -mdhc, -lysS, -CT058, -CT172, -hctB, -pbpB<br>6-gidA, -hemN, -pykF, -lysS, -CT058, -CT172, -hctB, -pbpB<br>7-gidA, -hemN, -pykF, -leuS, -CT058, -CT172, -hctB,-pbpB<br>8-gidA, -hemN, -lysS, -leuS, -CT058, -CT172, -hctB, -pbpB<br>9-gidA, -fumC, -mdhc, -lysS, -CT058, -CT172, -hctB, -pbpB<br>10-gidA, -fumC, -pykF, -lysS, -CT058, -CT172, -hctB, -pbpB<br>11-gidA, -fumC, -pykF, -leuS, -CT058, -CT172, -hctB, -pbpB<br>12-gidA, -fumC, -lysS, -leuS, -CT058, -CT172, -hctB, -pbpB<br>13-gidA, -mdhc, pykF, -lysS, -CT058, -CT172, -hctB, -pbpB<br>14-gidA, -mdhc, -lysS, -CT058, -CT144, -CT172, -hctB, -pbpB<br>15-gidA, -pykF, -lysS, -leuS, -CT058, -CT172, -hctB, -pbpB<br>16-gidA, -pykF, -lysS, -CT058, -CT144, -CT172, -hctB, -pbpB<br>17-gidA, -pykF, -leuS, -CT058, -CT144, -CT172, -hctB, -pbpB<br>18-gidA, -lysS, -leuS, -CT058, -CT144, -CT172, -hctB, -pbpB
